# Near-infrared photochromic miniprotein enables photoacoustic molecular imaging of acute inflammation and intracellular antigens

**DOI:** 10.1038/s41592-026-03255-y

**Published:** 2026-10-13

**Authors:** Olena S. Oliinyk, Yirui Xu, Jinhuan Luo, Kateryna O. Tokarchuk, Kyrylo Yu. Manoilov, Josie van de Klashorst, Matthew P. Eyring, Mu-en Wang, Ming Chen, Pengfei Song, John W. Hickey, Junjie Yao, Vladislav V. Verkhusha

**Affiliations:** 1 Department of Anatomy, Medicum, Faculty of Medicine, University of Helsinki, Helsinki, Finland; 2 Department of Biomedical Engineering, School of Engineering, Duke University, Durham, NC USA; 3 Department of Genetics and Gruss-Lipper Biophotonics Center, Albert Einstein College of Medicine, Bronx, NY USA; 4 Department of Pathology, School of Medicine, Duke University, Durham, NC USA

**Keywords:** Imaging, Molecular imaging, Sensors and probes, Protein design

## Abstract

Using near-infrared photoswitchable photochromic genetically encoded probes, differential photoacoustic tomography (PAT) enables deep-tissue imaging with high resolution and sensitivity; however, current bacteriophytochrome-based probes are bulky multidomain proteins exceeding 55 kDa, which constrains their use for designing PAT molecular reporters. Here we engineer rsmiRFPnano, a compact cyanobacteriochrome-derived photoswitchable PAT probe. With a molecular weight below 19 kDa, rsmiRFPnano efficiently incorporates biliverdin, exhibits high extinction coefficients in both photochromic states and demonstrates robust performance in live cells. We established rsmiRFPnano-based reporters for NF-κB and AP-1 pathways, which display low basal activity and strong activation-dependent responses. These reporters effectively visualized acute inflammation using noninvasive PAT in vivo, further validated through cellular-level spatial omics mapping. We then created photoswitchable nanobody probes, rsNIR-Fbs, stabilized upon antigen binding and rapidly degraded otherwise, enabling selective photoacoustic immuno-imaging of intracellular antigens in vivo. Together, these results establish rsmiRFPnano as an ultracompact and versatile platform for molecular PAT.

## Abstract (web summary)

A small, photoswitchable and genetically encodable protein operating in the near-infrared window is reported for photoacoustic tomography applications.

## Main

Photoacoustic tomography (PAT) combines the molecular contrast of optical absorption with the spatial resolution and penetration depth of ultrasound detection, enabling high-resolution imaging several centimeters deep in living tissues^1–4^. Photoacoustic signals can be generated by endogenous chromophores or exogenous contrast agents, among which genetically encoded probes offer distinct advantages, including precise spatial and temporal control of expression via tissue-specific promoters and sustained signal generation over extended periods in vivo^4^. Bacteriophytochrome (BphP)-derived genetically encoded probes have proven particularly effective, owing to their high extinction coefficients, absorption within the near-infrared (NIR) tissue transparency window, incorporation of the cellular chromophore biliverdin IXα (BV)^5^, and dual readout through both photoacoustic and fluorescent signals^6–8^.

A persistent challenge for conventional PAT is the strong background signal from endogenous absorbers, particularly due to the high concentration of hemoglobin in blood, which can easily overwhelm the weak signals from molecular probes. Differential PAT addresses this challenge by employing reversibly photoswitchable probes to generate ON- and OFF-state images. The images are processed to extract the differential signal, thereby suppressing the nonswitching background and enhancing detection sensitivity by up to three orders of magnitude^1,3,9^. This approach was first demonstrated using the bacterial phytochrome RpBphP1 from *Rhodopseudomonas* *palustris*^10^, which laid the conceptual foundation for the further development of reversibly photoswitchable BphP-based probes. BphPs absorb in the far-red to NIR range and undergo reversible photoconversion between two spectrally distinct absorbing states (Pr and Pfr)^11^. In transgenic mice with Cre-controlled BphP1 expression, differential PAT enabled quantitative mapping across multiple organs, including deep brain, embryos, virus-infected tissues and regenerating livers, at centimeter-scale depths in vivo^12,13^. Truncated variants, such as DrBphP-PCM, ReBphP-PCM and RpBphP1-PCM, have also been developed as photochromic PAT probes^14–16^.

Despite these advantages, BphPs are large multidomain proteins, which complicates their use as fusion tags or compact reporters for molecular imaging. Full-length RpBphP1 (~82 kDa) contains a photosensory core module (PCM) consisting of PAS–GAF–PHY domains and an additional effector domain^10^. Even truncated PCMs retain all three domains, remaining relatively large (>55 kDa) and dimeric, requiring the full PAS–GAF–PHY assembly for chromophore binding and reversible photoswitching.

To address the limitations of BphPs, we focused on cyanobacteriochromes (CBCRs), a diverse family of soluble photoreceptors from cyanobacteria. Similar to BphPs, CBCRs undergo reversible photoconversion; however, they require only a single GAF domain for chromophore binding, a compact architecture that has already proven suitable for engineering functional NIR fluorescent proteins (FPs)^17–20^. CBCRs also encompass multiple spectral lineages, including red-shifted verdin-specific CBCRs^21^. We therefore considered CBCRs a promising starting point for engineering small, NIR-absorbing photochromic probes for PAT.

However, the application of CBCRs in mammalian systems presents several challenges. Most CBCRs natively incorporate phycocyanobilin (PCB), which is abundant in cyanobacteria and plants but absent in mammalian cells, where BV serves as the available endogenous chromophore. Engineering CBCR GAF domains to bind BV often perturbs the native photocycle, leading to incomplete photoswitching, blue-shifted absorption or low extinction coefficients. We previously developed a miRFPnano series of NIR fluorescent proteins from the PCB-binding NpR3784 CBCR^17–20^. These proteins efficiently incorporate endogenous BV and exhibit high fluorescence brightness in mammalian cells, but the mutations required for BV binding disrupted the native photoconversion of NpR3784, making it unsuitable for reversibly photoswitchable probes. Attempts to adapt CBCR GAF domains for PAT have been reported earlier, with AM1_1557g2-derived proteins characterized in bacteria but not expressed in mammalian cells^22^.

Meanwhile, tracking endogenous intracellular targets and signaling pathways in deep tissues represents a particularly powerful application of PAT, enabling visualization of molecular processes and biological activities in living organisms. Nanobodies (Nbs), 15 kDa single-domain antigen-binding fragments from camelid heavy-chain antibodies, are especially well suited for this purpose because they bind intracellular antigens with high affinity and specificity. However, conventional Nbs are expressed independently of target presence, leading to the accumulation of excess unbound probes that generate background signal and obscure antigen-specific signals. To overcome this limitation, an approach converting Nbs into antigen-dependent fluorophores by inserting NIR FPs inside an Nb scaffold has been proposed^18,23^. These constructs, termed NIR-Fbs, are selectively stabilized upon antigen binding, whereas unbound molecules undergo rapid proteasome-dependent degradation. This design directly couples probe stability and fluorescence output to the presence of the target antigen, enabling high-contrast imaging of intracellular targets with minimal background.

Engineering antigen-stabilizable photochromic Nb probes would unlock a powerful class of molecular reporters for photoacoustic immuno-imaging deep in tissues. By combining antigen-dependent probe stabilization with photoswitching-based background suppression, such probes could enable selective detection of intracellular antigens, post-translationally modified proteins, and disease-associated targets within optically dense tissues. Achieving this capability requires ultracompact, single-domain photochromic proteins that can be genetically inserted into the Nb scaffold while preserving antigen recognition, intracellular stability and reversible photoswitching.

In addition to structural and functional imaging, an important frontier for PAT is the visualization of intracellular signaling pathways that govern processes such as inflammation and metabolic disorders. Photoacoustic probes based on organic dyes have been applied to monitor metabolites and enzyme activities^24^; however, genetically encoded reporters provide the added advantage of longitudinal, noninvasive monitoring of signaling dynamics within living tissues. Among these, transcription factors such as NF-κB and AP-1 play central roles in immune regulation, cell survival, and oncogenic transformation, and their dysregulation is closely associated with inflammation and cancer progression^25,26^.

Here, to develop a small photochromic probe for PAT, we focused on the third GAF domain of the JSC1_58120 CBCR from *Leptolyngbya* sp. (JSC1_58120g3: residues D537–A697), a representative of the verdin-specific CBCR lineage^21^. JSC1_58120g3 combines markedly red-shifted absorption spectra with intrinsic BV-binding capacity. Through several rounds of directed molecular evolution, we engineered JSC1_58120g3 into rsmiRFPnano, an 18.8 kDa NIR photochromic miniprobe that exhibits robust performance in mammalian cells and mice. We then used rsmiRFPnano to construct NF-κB and AP-1 transcriptional reporters, uniquely enabling differential PAT of acute inflammation signaling in vivo. This in vivo detection correlated well with the spatial omics profiling of inflammation biomarkers. Last, we engineered reversibly switchable nanobodies, rsNIR-Fbs, by inserting rsmiRFPnano into the Nb scaffold, thereby linking intracellular antigen recognition to antigen-dependent stabilization in live cells and in vivo.

## Results

### Engineering of rsmiRFPnano protein

To select a template for a BV-binding CBCR-based reversibly switchable PAT probe, we evaluated three GAF domains from the verdin-specific lineage: JSC1_58120g3, Mic7113_1903g4 and AFZ15460g4. Members of this lineage are characterized by red-shifted spectra and the ability to use 18^1^,18^2^-dihydrobiliverdin or BV as chromophores^21^. For efficient expression in mammalian cells, the genes were codon-optimized, and an N-terminal Met-Ala extension was added. The proteins were coexpressed in *Escherichia* *coli* together with heme oxygenase to synthesize BV. Among them, AFZ15460g4 exhibited poor BV incorporation, whereas JSC1_58120g3 and Mic7113_1903g4 provided a fluorescence readout, suggesting successful BV incorporation. Both of the latter recombinant proteins showed a fluorescent NIR-absorbing ground state and a nonfluorescent far-red-absorbing photoproduct. While both proteins were fluorescent in bacteria, only JSC1_58120g3 also produced faint but detectable fluorescence in mammalian cells. Based on this, JSC1_58120g3 was selected as the starting point for further molecular evolution.

**Fig. 1:**
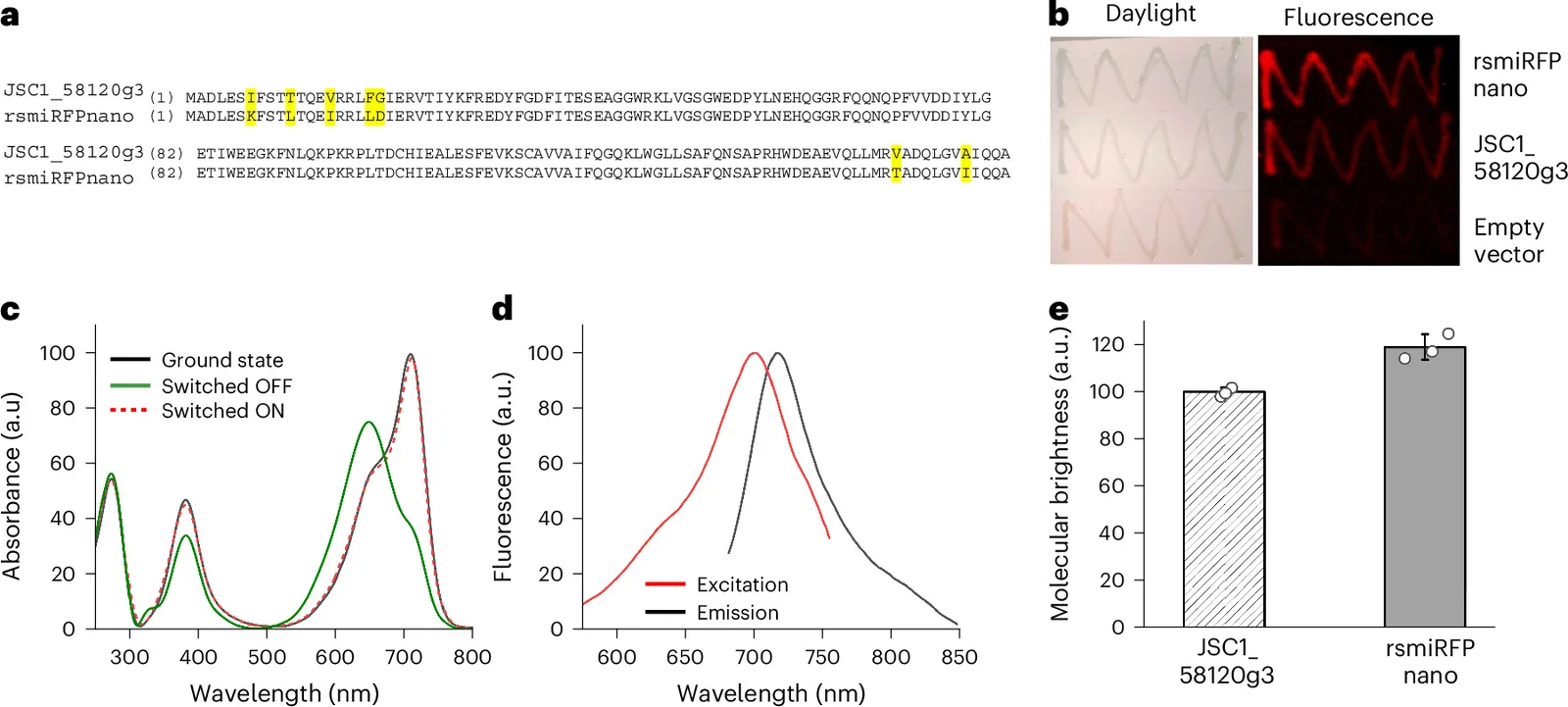
Engineering and spectral characterization of rsmiRFPnano. **a**, Amino acid sequence alignment of the parental JSC1_58120g3 and rsmiRFPnano. **b**, Comparison of JSC1_58120g3 and rsmiRFPnano expressed in *E.* *coli* cells. For fluorescence imaging, a 685/20 nm excitation filter and a 730 nm long-pass emission filter were used. **c**, Absorbance spectra of rsmiRFPnano in the ground NIR-absorbing state (black line), after illumination with 740 nm light (green line; switched-OFF far-red-absorbing state), and after subsequent illumination with 660 nm light (red line; switched-ON NIR-absorbing state). **d**, Fluorescence excitation and emission spectra of rsmiRFPnano. **e**, Comparison of the molecular brightness of JSC1_58120g3 and rsmiRFPnano. Data are presented as mean ± s.d., *n* = 3 independent experiments. Source Data

As mutations in the BV-binding pocket often disrupt the photocycle, we adopted a strategy that targeted structural regions outside the pocket. In our previous work on constitutively fluorescent miRFP670nano3 (ref. ^18^), we identified critical mutations outside the BV-binding site that improved protein performance. Guided by this, we applied iterative saturation mutagenesis to the amino acid residues in the N- and C-terminal helices that face one another in the protein structure (PDB ID 7LSC)^20^. Four sequential rounds of mutagenesis targeted residues F19 and E145, V15 and V152, T11 and L156, and I7 and A159, respectively (Supplementary Fig. 1). In each round, libraries were first screened in bacteria to identify clones that preserved fluorescence and reversible photoconversion. Selected variants were then evaluated in transiently transfected mammalian cells. The best-performing clone, selected based on improved fluorescence brightness reflecting efficient intracellular performance, including BV incorporation, stability and expression, was used as a template for the subsequent mutagenesis round. This directed molecular evolution yielded rsmiRFPnano, a reversibly photoswitchable protein consisting of 163 amino acid residues (18.8 kDa) and carrying seven amino acid substitutions relative to wild-type JSC1_58120g3 (numbering according to rsmiRFPnano sequence): I7K, T11L, V15I, F19L, G20D, V152T and A159I (Fig. 1a).

### Characterization of recombinant rsmiRFPnano

rsmiRFPnano was characterized in comparison to its parental protein (Fig. 1). The design strategy was directed toward retaining the photochromism of the wild-type JSC1_58120g3 while improving expression efficiency and BV binding. Both proteins exhibited an NIR–far-red photocycle, indicating that the reversible switching mechanism was preserved (Fig. 1c and Supplementary Fig. 2). The ground state of both proteins was characterized by a major absorbance Q-peak at 715 nm and a minor Soret band at 385 nm, typical of tetrapyrrole-binding proteins. Illumination with 740 nm light converted the proteins into a far-red-absorbing photoproduct with a Q-peak at 650 nm and a corresponding Soret band at 385 nm. Subsequent exposure to 660 nm illumination restored the NIR-absorbing state, demonstrating the efficient reversibility.

JSC1_58120g3 exhibited relatively high molar extinction coefficients of 80,000 M^−1^ cm^−1^ for the NIR-absorbing state and 82,000 M^−1^ cm^−1^ for the far-red-absorbing photoproduct. Notably, rsmiRFPnano showed increased extinction coefficients of 85,000 and 88,000 M^−1^ cm^−1^, respectively, indicating that the engineered variant not only preserved but enhanced the light-absorption properties of the parental protein (Supplementary Table 1). Both proteins exhibited fluorescence in the NIR-absorbing state, whereas the far-red-absorbing photoproducts were nonfluorescent. Their fluorescence spectra were nearly identical, with excitation maxima at 715 nm and emission maxima at 732 nm (Fig. 1d and Supplementary Fig. 3); however, in BV-producing *E.* *coli*, rsmiRFPnano displayed markedly stronger NIR fluorescence compared to JSC1_58120g3 (Fig. 1b). Quantitative analysis of recombinant proteins further showed that the molecular brightness of rsmiRFPnano was ~1.2-fold higher than that of JSC1_58120g3 (Fig. 1e and Supplementary Table 1).

rsmiRFPnano exhibited p*K*a values of 4.2 and 4.3 for the ground and photoswitched states, respectively (Supplementary Fig. 4 and Supplementary Table 1), indicating stable spectral properties across the physiological pH range. Native polyacrylamide gel electrophoresis (PAGE) analysis showed that rsmiRFPnano migrated as a single fluorescent species without detectable aggregates (Supplementary Fig. 5), consistent with monomeric behavior expected for a compact single-domain GAF protein.

### Photoacoustic characterization of rsmiRFPnano in vitro

**Fig. 2:**
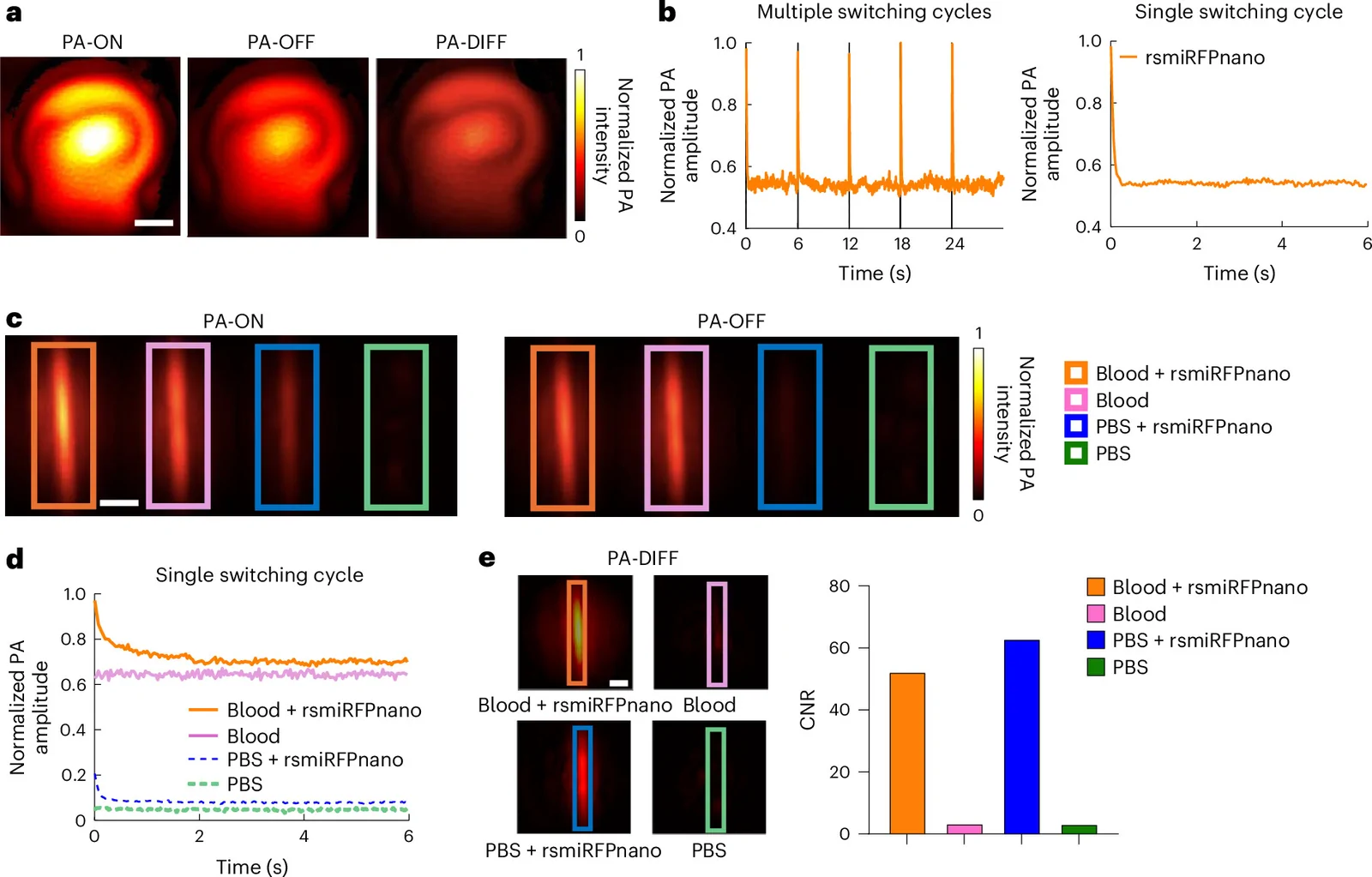
Photoacoustic characterization of purified rsmiRFPnano and rsmiRFPnano-expressing bacteria. **a**, PA images of *E.* *coli* cells expressing rsmiRFPnano in the ON and OFF states, and the resulting differential PA image (PA-DIFF = PA-ON – PA-OFF). **b**, Dynamic PA signal changes over five consecutive photoswitching cycles (left) and the corresponding averaged temporal profile over a single cycle (right). **c**, PA-ON and PA-OFF images of samples containing blood mixed with purified rsmiRFPnano protein (17 µM), pure blood, PBS with purified rsmiRFPnano protein (17 µM) and pure PBS as a control. **d**, Comparison of dynamic PA signal changes over a single switching cycle for the four samples shown in **c**. **e**, PA-DIFF images and corresponding calculated CNR values for the four samples. CNR was calculated as (*I*_*S*_ – *I*_*B*_)/*σ*_*B*_, where *I*_*S*_ is the mean PA-DIFF signal within the boxed region of interest, *I*_*B*_ is the mean signal in a probe-free background region, and *σ*_*B*_ is the s.d. of the background signal. Scale bars, 1 mm. Source Data

To evaluate rsmiRFPnano as a photochromic contrast agent for PAT, we characterized its photoswitching performance in vitro using rsmiRFPnano-expressing bacteria and purified rsmiRFPnano protein samples in various background environments (Fig. 2). Under alternating illumination, *E.* *coli* expressing rsmiRFPnano exhibited robust and reversible modulation of the photoacoustic PA signals between the ON (PA-ON) and OFF (PA-OFF) states (Fig. 2a and Supplementary Fig. 6). Temporal subtraction of the PA-OFF image from the PA-ON image generated a differential PA image (PA-DIFF), which efficiently suppressed nonswitching background signals and highlighted the spatial distribution of rsmiRFPnano. The PA signals followed a consistent and reversible dynamic profile over multiple photoswitching cycles, demonstrating stably reversible photoswitching of rsmiRFPnano expressed in bacteria (Fig. 2b).

To assess the impact of hemoglobin-dominated background absorption on photoswitching detectability, purified rsmiRFPnano protein (17 µM) was imaged in whole blood and in phosphate-buffered saline (PBS), together with pure blood and pure PBS controls in a transparent silicone tube (Fig. 2c). Dynamic PA measurements revealed clear photoswitching-induced signal modulation in samples containing rsmiRFPnano, whereas pure blood and PBS showed no photoswitching behavior (Fig. 2d). All PA signals were globally normalized to the maximum signal amplitude across samples to enable direct comparison.

To quantitatively evaluate the detection sensitivity of differential PAT, we calculated the contrast-to-noise ratio (CNR) from PA-DIFF images (Fig. 2e and Supplementary Table 2). Despite strong hemoglobin absorption, rsmiRFPnano in whole blood exhibited a high PA-DIFF CNR that was comparable to that obtained for rsmiRFPnano in PBS, indicating effective suppression of blood signals by photoswitching differentiation. The effective noise-equivalent detection sensitivity for rsmiRFPnano was ~0.33 µM with whole blood as the background, estimated as the probe concentration at which the differential PA CNR equals 1, assuming a linear dependence of CNR on concentration. In contrast, whole blood and PBS yielded low PA-DIFF CNRs, confirming the absence of photoswitching-derived contrast from endogenous absorbers (for example, hemoglobin and water) (Supplementary Table 2).

Together, these results demonstrate that rsmiRFPnano enables high-sensitivity differential PAT even in the presence of a strong hemoglobin background, achieving high detectability comparable to that in a background-free environment and supporting its suitability for differential PAT in complex biological settings.

### Characterization of rsmiRFPnano protein in live mammalian cells

A key requirement for genetically encoded PAT probes is robust performance in mammalian cells, as in vivo applications cannot be achieved without efficient chromophore incorporation and high expression. While the parental JSC1_58120g3 exhibited favorable photochemical properties in bacteria, including reversible switching and strong absorbance, its performance in mammalian cells was limited. Therefore, improving expression and brightness in mammalian systems represented the main challenge in developing a functional CBCR GAF domain-based probe for PAT.

**Extended Data Fig. 1:**
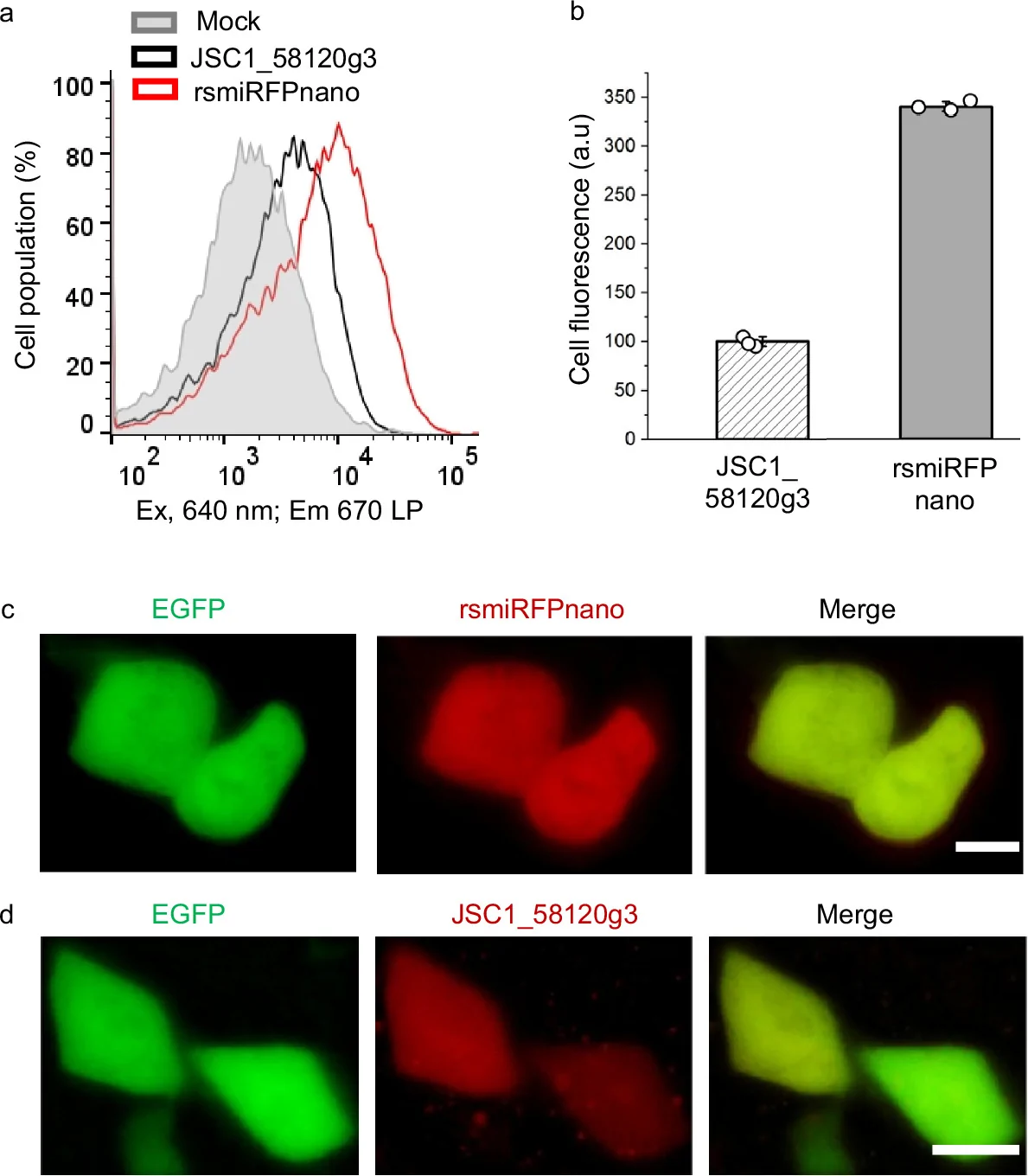
Comparison of the performance of parental JSC1_58120g3 and rsmiRFPnano in mammalian cells. (**a**) Fluorescence intensity distribution in live HEK293T cells transiently transfected with JSC1_58120g3 or rsmiRFPnano. (**b**) Quantification of the data shown in (a). Data are presented as mean ± s.d., *n* = 3 independent transfection experiments. (**c, d**) Fluorescence images of live HEK293T cells co-transfected with EGFP and either rsmiRFPnano (c) or JSC1_58120g3 (d). Representative images from 3 independent experiments are shown. Scale bars, 10 μm. For imaging of rsmiRFPnano or JSC1_58120g3 and EGFP, excitation/emission filter sets of 685/20 nm-725/40 nm and 485/20 nm-525/30 nm were used, respectively. Source Data

To evaluate cellular performance, we compared rsmiRFPnano with JSC1_58120g3 in transiently transfected HEK293T cells. Flow cytometry revealed a 3.5-fold increase in fluorescence intensity for rsmiRFPnano relative to the parental protein (Extended Data Fig. 1a,b). This substantial improvement demonstrates that directed evolution not only preserved the favorable photocycle of the CBCR template but also overcame its poor performance in mammalian cells, thereby enabling the application of rsmiRFPnano for in vivo PAT. Fluorescence microscopy further confirmed a bright and homogeneous signal from rsmiRFPnano, in contrast to the weak fluorescence of cells expressing JSC1_58120g3 (Extended Data Fig. 1c,d).

To further characterize rsmiRFPnano in mammalian cells, we evaluated its accumulation kinetics and intracellular stability (Supplementary Figs. 7 and 8). In a doxycycline-inducible Tet-On expression system, rsmiRFPnano exhibited fluorescence accumulation kinetics comparable to EGFP under the same experimental conditions (Supplementary Fig. 7). The apparent half-time of fluorescence accumulation was ~27 h for rsmiRFPnano and ~34 h for EGFP. In cycloheximide chase experiments, rsmiRFPnano showed an apparent intracellular fluorescence half-life of ~18 h in HEK293T cells (Supplementary Fig. 8).

These results establish rsmiRFPnano as a compact CBCR-derived probe that combines reversible photoswitching with robust functionality in mammalian systems, an essential requirement for deep-tissue PA imaging applications.

### Design of rsmiRFPnano-based reporters for NF-κB and AP-1 pathways

The transcription factors NF-κB and AP-1 are central regulators of cellular responses to stress, infection and tissue damage. In normal physiology, they orchestrate processes such as immune activation, cell proliferation, survival and differentiation. Dysregulated NF-κB and AP-1 signaling is implicated in chronic inflammatory diseases and diverse cancers, where persistent activation promotes tumor growth, angiogenesis, metastasis and resistance to apoptosis^25–27^. Given their dual roles in maintaining homeostasis and driving disease progression, NF-κB and AP-1 remain critical molecular readouts for both basic research and translational applications.

**Fig. 3:**
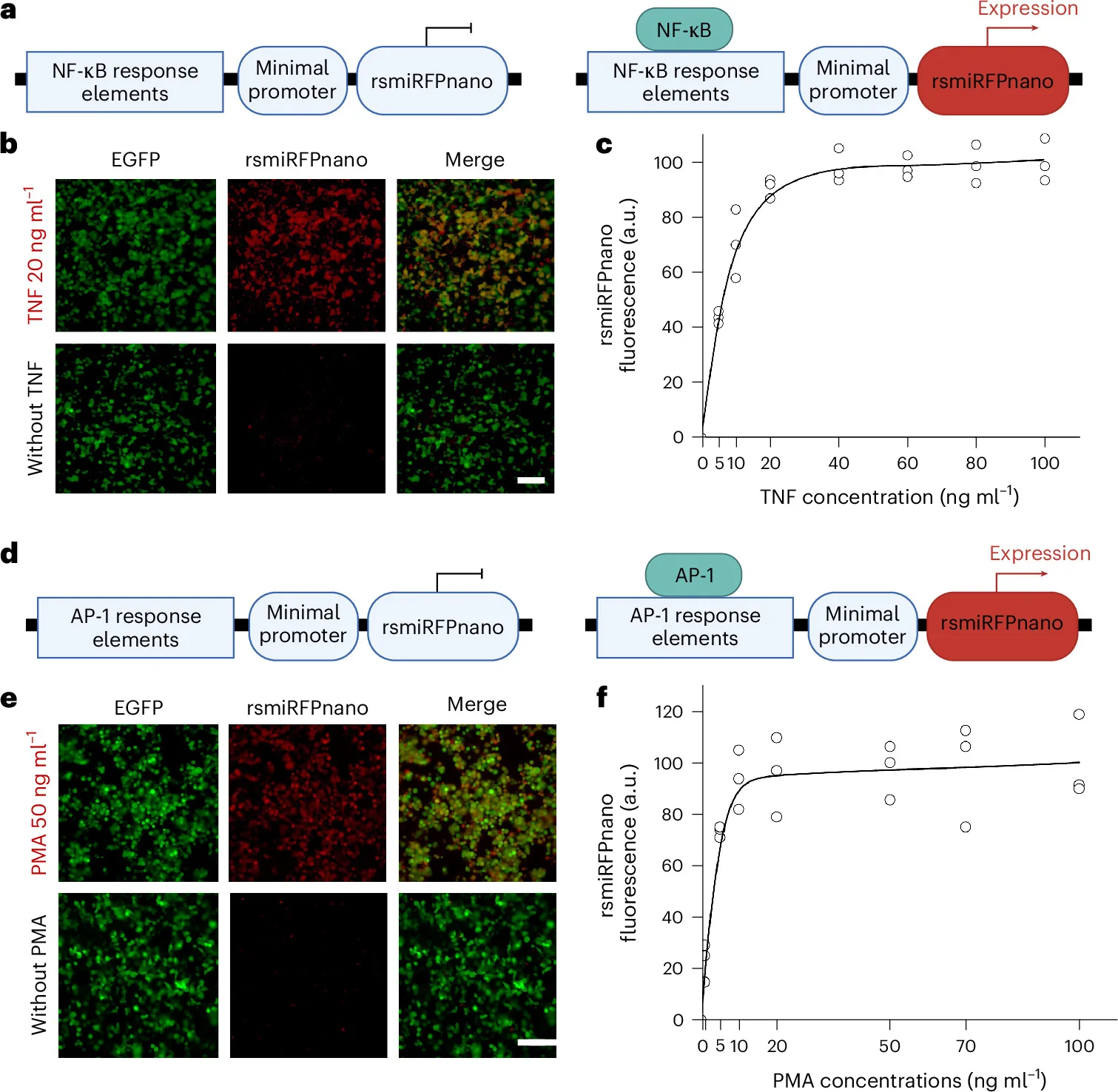
Performance of rsmiRFPnano-based NF-κB and AP-1 inflammation reporters in cells. **a**, Schematic of the rsmiRFPnano-based NF-κB reporter. **b**, Fluorescence images of HEK293T cells transfected with the NF-κB reporter and co-transfected with pEGFP-N1 (1:10). Cells were either stimulated with TNF (20 ng ml⁻^1^, top row) or left untreated (without, bottom row). **c**, TNF dose–response of the NF-κB reporter measured by flow cytometry. Data are presented as mean ± s.d. for *n* = 3 transfection experiments. **d**, Schematic of the rsmiRFPnano-based AP-1 reporter. **e**, Fluorescence images of HEK293T cells transfected with the AP-1 reporter and co-transfected with pEGFP-N1 (1:10). Cells were either stimulated with PMA (50 ng ml⁻^1^, top row) or left untreated (without PMA, bottom row). **f**, PMA dose–response of the AP-1 reporter measured by flow cytometry. Data are presented as mean ± s.d. for *n* = 3 transfection experiments. Fluorescence imaging was performed using excitation/emission filter sets of 685/20 nm−725/40 nm for rsmiRFPnano and 485/20 nm−525/30 nm for EGFP. Representative images from three independent experiments are shown. Scale bars, 100 µm. Source Data

To enable PAT-compatible readout of transcriptional activity in live cells, rsmiRFPnano-based reporters for NF-κB and AP-1 were generated (Fig. 3). Each construct incorporated tandem consensus response elements upstream of a minimal promoter driving rsmiRFPnano expression (schematics in Fig. 3a,d). The NF-κB reporter contained five response elements, whereas the AP-1 reporter was generated with six AP-1 consensus sites. Upon pathway activation, NF-κB and AP-1 bind to their respective elements and initiate transcription of the downstream rsmiRFPnano gene.

Reporter plasmids were transiently expressed in HEK293T cells together with pEGFP-N1 (1:10) to mark transfected cells. Because rsmiRFPnano is intrinsically fluorescent, reporter output could be quantified directly by NIR fluorescence microscopy and flow cytometry. Under basal conditions, cells exhibited minimal NIR fluorescence signals, indicating low promoter leakiness. Upon tumor necrosis factor (TNF) stimulation (20 ng ml^−1^), the NF-κB reporter was robustly induced, producing bright rsmiRFPnano fluorescence in EGFP-positive cells (Fig. 3b). Flow cytometry revealed a dose-dependent activation, with detectable induction at 1 ng ml^−1^ TNF and a near-maximal response at ≥5 ng ml^−1^ (Fig. 3c). Similarly, phorbol 12-myristate 13-acetate (PMA) stimulation (50 ng ml^−1^) triggered strong induction of the AP-1 reporter (Fig. 3e), with a graded dose–response confirmed by flow cytometry (Fig. 3f). Nonstimulated controls remained at near-background levels, underscoring the low basal output of the reporters.

Together, these results establish rsmiRFPnano as a sensitive, bright, low-leakage NIR transcriptional reporter for probing NF-κB and AP-1 activity in mammalian cells, while preserving the reversible photoswitching required for downstream photoacoustic molecular imaging of the corresponding inflammatory pathways.

### PAT of rsmiRFPnano NF-κB and AP-1 reporters in deep tissues

To evaluate the in vivo performance of rsmiRFPnano-based inflammation reporters of NF-κB or AP-1, we expressed the corresponding reporters in the livers of two separate groups of mice via hydrodynamic transfection and subsequently administered systemic lipopolysaccharide (LPS) to induce acute inflammation (Supplementary Fig. 9a). PA imaging was performed 72 h before LPS injection to establish baseline conditions, and subsequently from 0 to 120 h post-LPS treatment to longitudinally track changes in transcriptional activity.

**Fig. 4:**
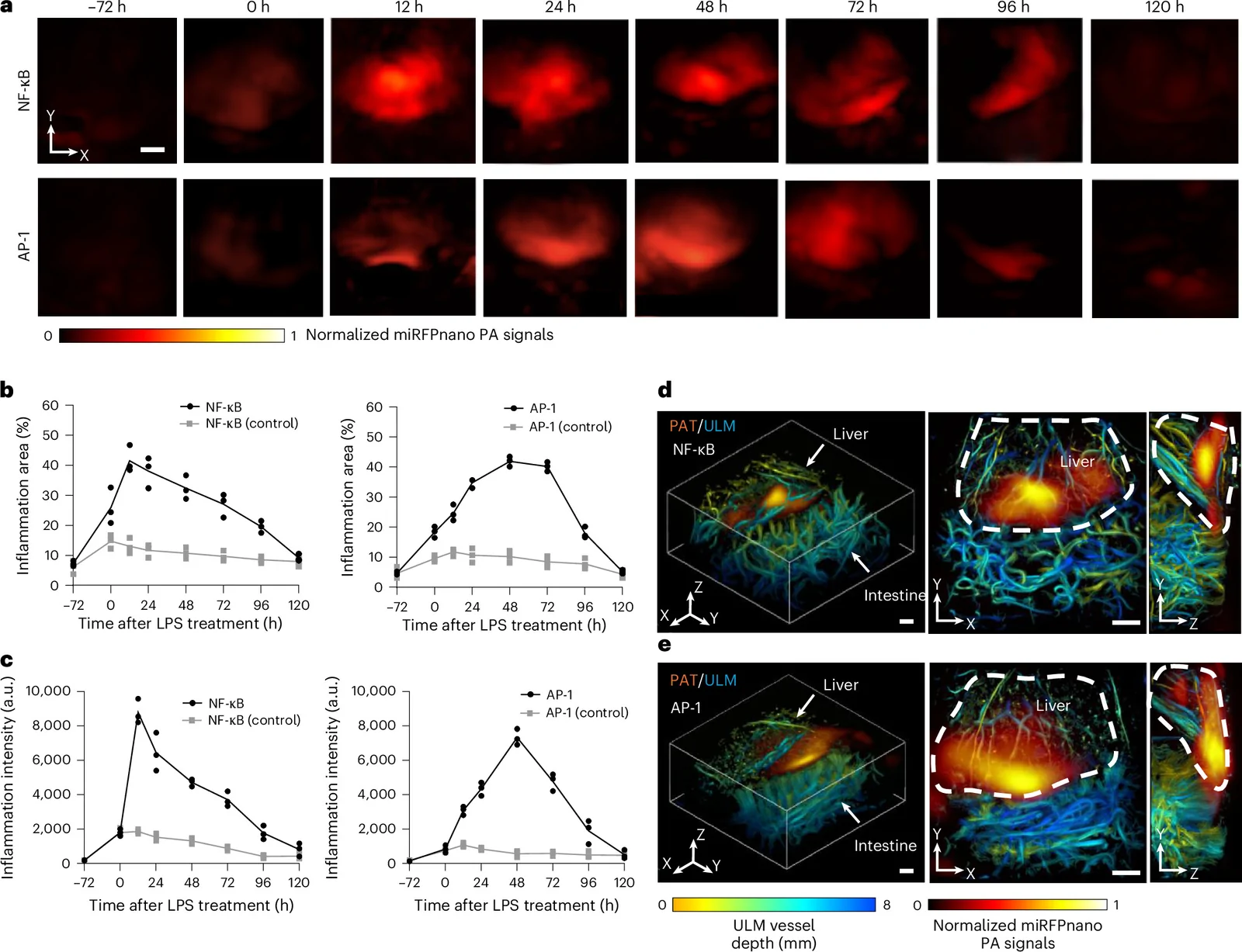
Photoacoustic imaging of rsmiRFPnano-based NF- κB and AP-1 reporters in mice upon LPS treatment. **a**, Representative differential PAT images (PA-DIFF) showing rsmiRFPnano expression in the liver at different time points before and after LPS treatment (top row, NF-κB reporter; bottom row, AP-1 reporter). Scale bar, 2 mm. **b**, Quantification of inflammation area over time for the NF-κB and AP-1 reporter groups (*n* = 3 mice per group) in the LPS-treated and PBS-treated control groups. The inflammation area was computed as the area percentage with the differential PA signals above the noise floor. **c**, Quantification of averaged differential PA signals over time for the NF-κB and AP-1 reporter groups in LPS-treated and control groups. The differential PA signals were averaged over the inflammation area. **d**,**e**, Representative three-dimensional hybrid differential PAT and ULM images acquired at 48 h after LPS treatment from the NF-κB (**d**) and AP-1 reporter (**e**) groups, showing the co-registered PAT/ULM volume renderings (left) and orthogonal maximum-intensity projection images (middle and right). Differential PA signals corresponding to rsmiRFPnano expression (hot) are overlaid on the liver microvasculature obtained by ULM (Jet). Scale bars, 2 mm. Source Data

Representative ON-state and OFF-state PA images revealed strong photoacoustic signals from both the liver and surrounding intestinal regions, reflecting the substantial endogenous background signals present in vivo (Supplementary Figs. 10 and 11). In contrast, differential processing selectively isolated the rsmiRFPnano signals expressed in the liver while suppressing nonswitching background signals. As a result, differential PAT provided high-contrast visualization of hepatic reporter expression and enabled specific detection of inflammation-associated transcriptional activity in vivo (Fig. 4a).

Following LPS treatment, both the NF-κB and AP-1 reporters exhibited time-dependent increases in differential PA signal, indicating inflammation-associated transcriptional activation. At later time points, the differential PA signal gradually decreased as the inflammatory response subsided. A consistent trend was also observed in whole-body fluorescence imaging performed on the same animals, with liver-localized fluorescence signals evolving over time after LPS treatment (Supplementary Fig. 9b). Notably, NF-κB and AP-1 reporters displayed different activation kinetics, with the NF-κB reporter showing rapid induction and showing maximal signals at 12 h post-LPS treatment, whereas the AP-1 reporter exhibited a more gradual response, peaking at 48 h (Fig. 4a). Similar temporal differences between rapid NF-κB activation and delayed AP-1 activation have been reported previously in liver injury models^28^. These temporally resolved differences demonstrate the ability of the noninvasive PA imaging approach to distinguish pathway-specific inflammatory dynamics in vivo.

Quantitative analysis further characterized these reporters using two metrics: inflammation area, defined as the fraction of liver area with differential PA signals (Fig. 4b), and mean differential PA signals within the liver region of interest, reflecting the magnitude of reporter expression (Fig. 4c). Together, these measures captured both the spatial extent and the strength of reporter activation. To distinguish LPS-induced reporter activation from potential effects of hydrodynamic transfection alone, we performed additional control experiments in which mice received the same NF-κB or AP-1 reporter constructs but were treated with PBS instead of LPS (Supplementary Figs. 12–14). In these control animals, weak reporter signals were detected at early time points, consistent with transient liver stress associated with hydrodynamic transfection; however, neither fluorescence imaging nor differential PAT showed evidence of the sustained activation observed in the LPS-treated groups. The PBS-treated groups had peak inflammation intensities that were fivefold and sevenfold weaker than those of the LPS-treated groups with NF-κB or AP-1 reporters, respectively. These findings indicate that the longitudinal reporter activation observed following LPS treatment primarily reflects inflammation-induced NF-κB and AP-1 signaling rather than transfection-related effects alone.

### PAT integration with super-resolution ultrasound localization microscopy

**Extended Data Fig. 2:**
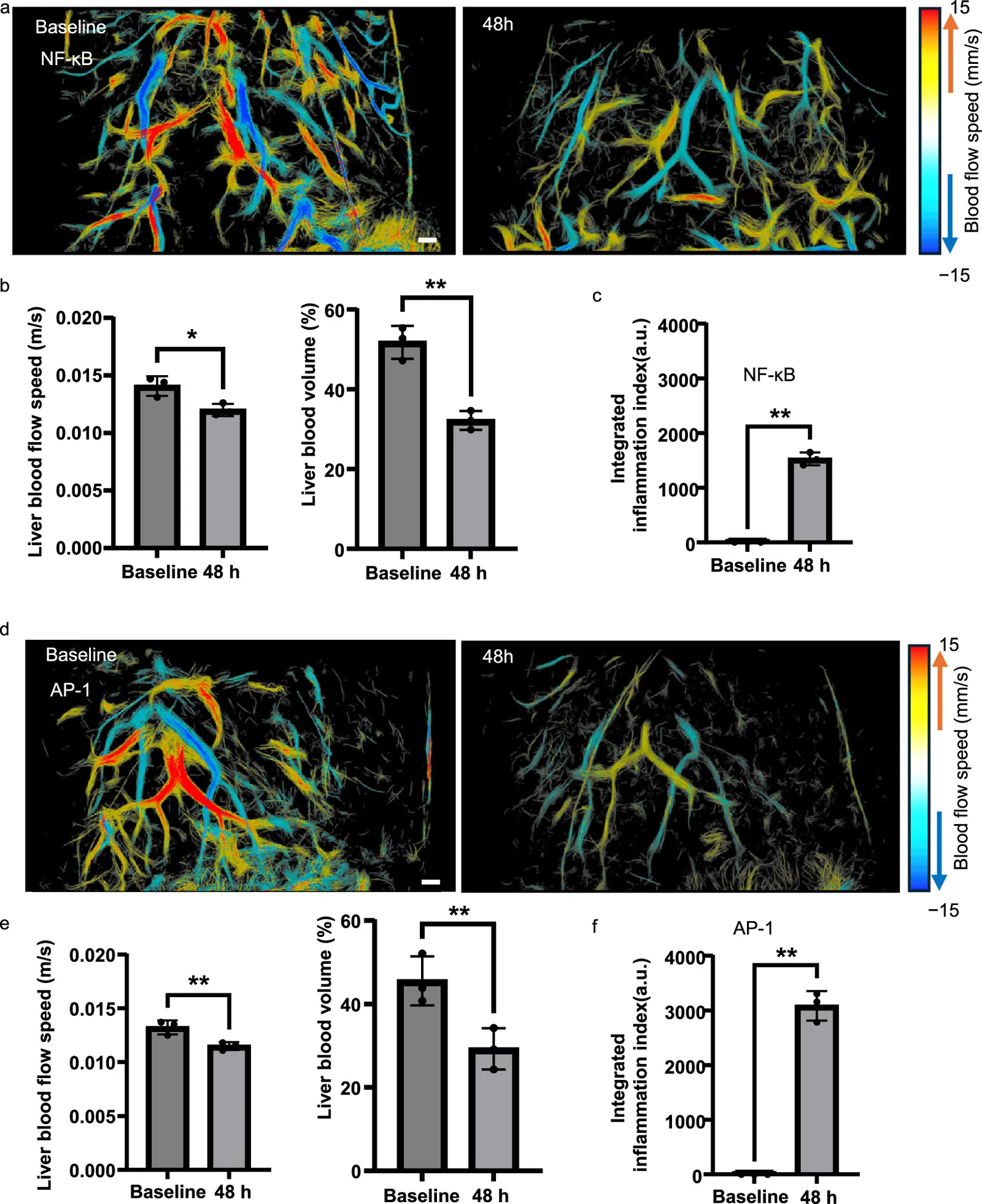
Functional and molecular quantification of liver inflammation by hybrid PAT and ULM. (**a**) Representative flow-encoded ULM images of the liver blood flow before and 48 h after the lipopolysaccharide (LPS) treatment in the NF-κB reporter groups, showing the reduced blood flow induced by inflammation. Images are representative of *n* = 3 mice. Scale bar, 1 mm. (**b-c**) Quantification of the averaged (b) liver blood-flow speed, blood volume, and (c) inflammation index as reported by differential PAT of the NF-κB reporter. The inflammation index was computed as the total differential PAT signals of the NF-κB reporter in the inflammation area. Data are presented as mean ± s.d.; *n* = 3 mice (biological replicates, that is independent animals). Statistical significance was assessed using paired two-tailed parametric t-tests. In (b), ^*^P = 0.0229 (blood-flow speed) and ^*^P = 0.0031 (blood volume); in (c), ^*^P = 0.0019. (**d**) Representative flow-encoded ULM images of the liver blood flow before and 48 h after the LPS treatment in the AP-1 reporter groups, showing the reduced blood flow induced by inflammation. Images are representative of *n* = 3 mice. Scale bar, 1 mm. (**e-f**) Quantification of the averaged (e) liver blood-flow speed, blood volume, and (f) inflammation index as reported by differential PAT of the AP-1 reporter. The inflammation index was computed as the total differential PAT signals of the AP-1 reporter in the inflammation area. Data are presented as mean ± s.d.; *n* = 3 mice (biological replicates, that is independent animals). Statistical significance was assessed using paired two-tailed parametric t-tests. In (e), *P* = 0.0091 (blood-flow speed) and *P* = 0.0032 (blood volume); in (f), *P* = 0.0026. Source Data

In addition to differential PAT, we also performed super-resolution ultrasound localization microscopy (ULM) using the same imaging system (Extended Data Fig. 2 and Supplementary Fig. 15). The hybrid differential PAT and ULM system has been previously reported by our teams^12,29^. Representative hybrid PAT/ULM renderings enabled three-dimensional co-registered visualization of NF-κB and AP-1 reporter activation by PAT together with the hepatic microvasculature resolved by ULM (Fig. 4d,e). The ULM-based blood-flow maps further revealed pronounced inflammation-associated hemodynamic alterations in the liver (Extended Data Fig. 2a,d). Quantitative analysis demonstrated significant decreases in both liver blood-flow speed and blood volume at 48 h post-LPS treatment compared to baseline conditions (Extended Data Fig. 2b,e). To quantify the overall hepatic inflammatory response, an integrated inflammation index was calculated as the product of inflammation area and mean differential PA signal intensity. This integrated metric revealed a pronounced increase in inflammatory burden at 48 h post-LPS relative to baseline, coinciding with the reductions in liver blood-flow speed and blood volume (Extended Data Fig. 2c,f).

Collectively, these results demonstrate that rsmiRFPnano reporters enable noninvasive, longitudinal, and spatially resolved tracking of liver inflammation by differential PAT and ULM, providing complementary molecular and hemodynamic readouts of the inflammatory response in vivo.

### Spatial omics profiling of LPS-induced liver inflammation

**Fig. 5:**
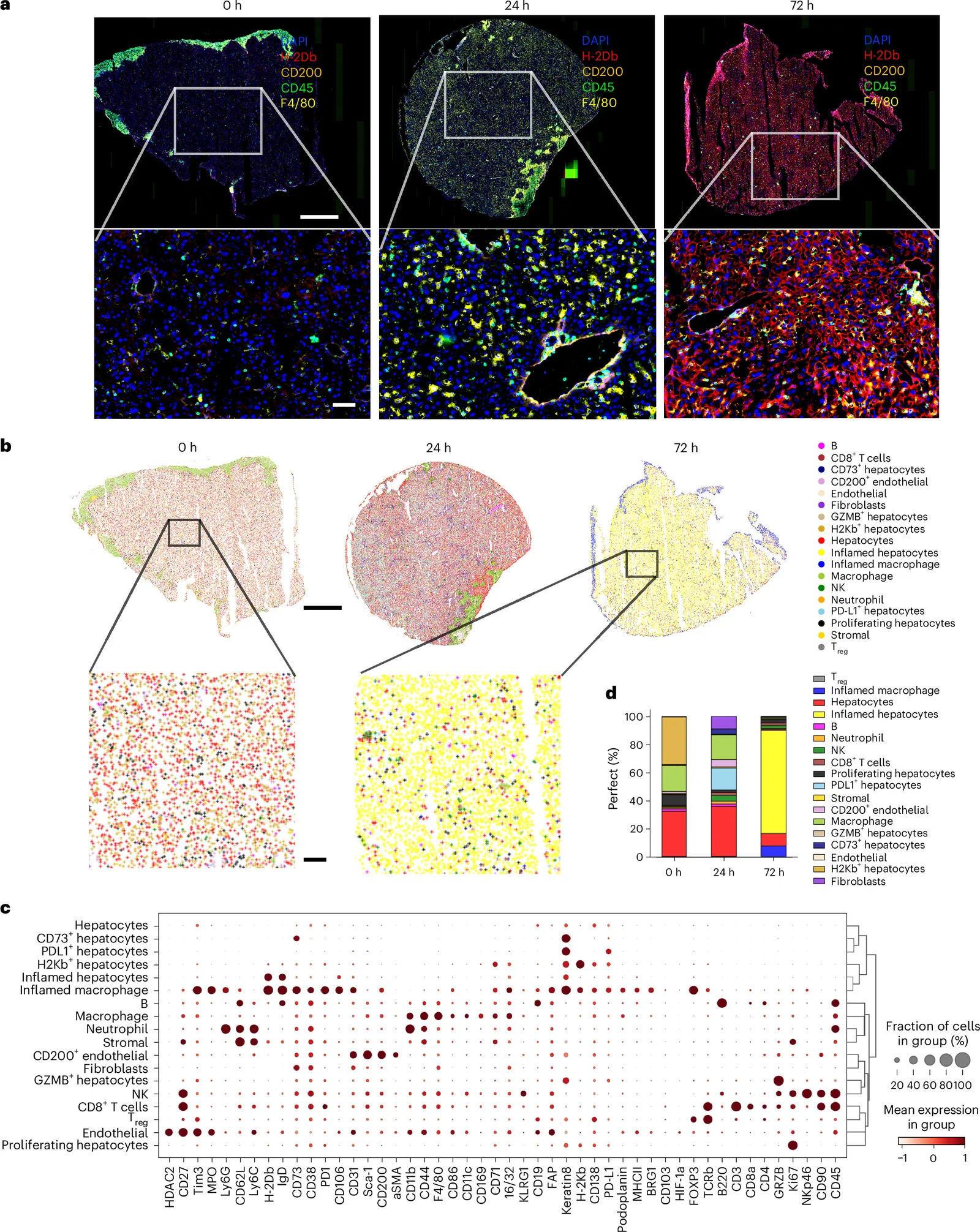
Spatiotemporal profiling of LPS-induced liver inflammation by CODEX imaging. **a**, Representative CODEX multiplexed immunofluorescence images of mouse liver sections collected at 0, 24 and 72 h following LPS treatment, showing selected biomarkers from the 50-antibody panel, including 4,6-diamidino-2-phenylindole (DAPI) (nuclei), H-2Db (MHC class I), CD200, CD45 (pan-immune cells) and F4/80 (macrophages). Whole-tissue views (top row) and corresponding close-up regions (bottom row) are shown. CODEX imaging was performed on one mouse per time point (*n* = 1 biological replicate). **b**, Spatial maps of single-cell-resolved cell-type annotations across liver sections at 0, 24 and 72 h post-LPS treatment. Cells were segmented and classified by unsupervised clustering based on protein expression from the full CODEX antibody panel. Major epithelial, stromal and immune cell populations are shown. The close-up regions (bottom row) at 0 and 72 h post-LPS treatment highlight the significant increase in inflamed hepatocytes (shown in yellow) at 72 h. **c**, Dot plot showing expression of selected lineage and functional markers across annotated cell populations. Dot size represents the fraction of cells within each population expressing the marker, and color intensity indicates mean normalized expression. **d**, Relative abundance of annotated cell populations at 0, 24 and 72 h following LPS treatment. Stacked bar plots show the percentage contribution of each cell type to the total cellular composition at each time point. Scale bars, 500 μm (whole-tissue views) and 50 μm (close-up).

We next performed multiplexed spatial proteomics and single-cell mapping (CODEX) on mouse liver samples collected at 0, 24 and 72 h following LPS treatment, which independently characterized inflammation-associated cellular changes at single-cell resolution (Fig. 5)^30,31^. A 50-antibody panel was used to simultaneously profile major epithelial, stromal, and immune cell populations, including hepatocytes, endothelial cells, macrophages and lymphocyte subsets, together with functional markers associated with inflammatory activation, for example, Ki67, PD-1 and H-2Db (Fig. 5a and Supplementary Fig. 16 and 17).

Even when examining a subset of these markers, pronounced temporal changes in liver inflammatory status were observed (Fig. 5a). H-2Db, a major histocompatibility complex class I molecule inducible by inflammatory cytokines, showed minimal expression at baseline (0 h), with signal largely restricted to endothelial cells. At 24 h after LPS treatment, H-2Db expression increased modestly, whereas by 72 h it was robustly upregulated throughout the tissue, including widespread expression in hepatocytes, consistent with a sustained tissue-wide inflammatory activation state (Fig. 5a,c).

In contrast, markers associated with immune cell infiltration displayed a distinct temporal pattern. F4/80^+^ macrophages and CD45^+^ hematopoietic immune cells were sparsely detected at baseline and were primarily localized near vascular structures. Both populations increased markedly by 24 h following LPS treatment, consistent with robust recruitment and activation of innate immune cells during the acute inflammatory phase. By 72 h, the relative abundance of CD45^+^ and F4/80^+^ cells was reduced compared to 24 h, indicating partial attenuation of immune cell infiltration at later time points (Fig. 5a,d).

To further quantify these dynamics, we performed single-cell segmentation, unsupervised clustering, and cell-type annotation based on protein expression across the full CODEX antibody panel (Fig. 5b and Supplementary Fig. 18). We used several computational tools for large spatial omics imaging datasets^30,32–42^, including the integration framework MaxFuse^34^, image processing and registration tools, deep-learning algorithms such as STELLAR^33^, and hierarchical cellular neighborhood analysis^37^. Spatial maps of annotated cell populations revealed substantial intra-tissue heterogeneity and indicated that immune cell populations peak at 24 h, whereas inflamed hepatocytes constitute the dominant cell population at 72 h (Fig. 5b–d). These spatial omics results provide independent, cell-resolved evidence of the spatiotemporal progression of LPS-induced liver inflammation and correlated well with the differential PAT results observed in vivo.

### Engineering of antigen-stabilizable photochromic NIR-Fbs

To engineer antigen-stabilizable photochromic Nb probes, we incorporated rsmiRFPnano as an internal fusion within a Nb scaffold using a structurally defined NIR-Fb architecture^18,23,43^. This design extends the previously established antigen-stabilizable Nb approach by incorporating the reversibly switchable rsmiRFPnano. Based on the design for antigen-dependent Nb stabilization^18,23,43^, rsmiRFPnano was inserted into a loop within the third framework region (FR3), distal from the antigen-binding interface. This position is between Ser65 and Val66 residues in the Nb_GFP_ (Supplementary Fig. 19). Insertions at this position using Gly_4_Ser flexible linkers preserve antigen recognition by complementarity-determining regions (CDRs), while introducing controlled destabilization of the unbound Nb^18^.

**Fig. 6:**
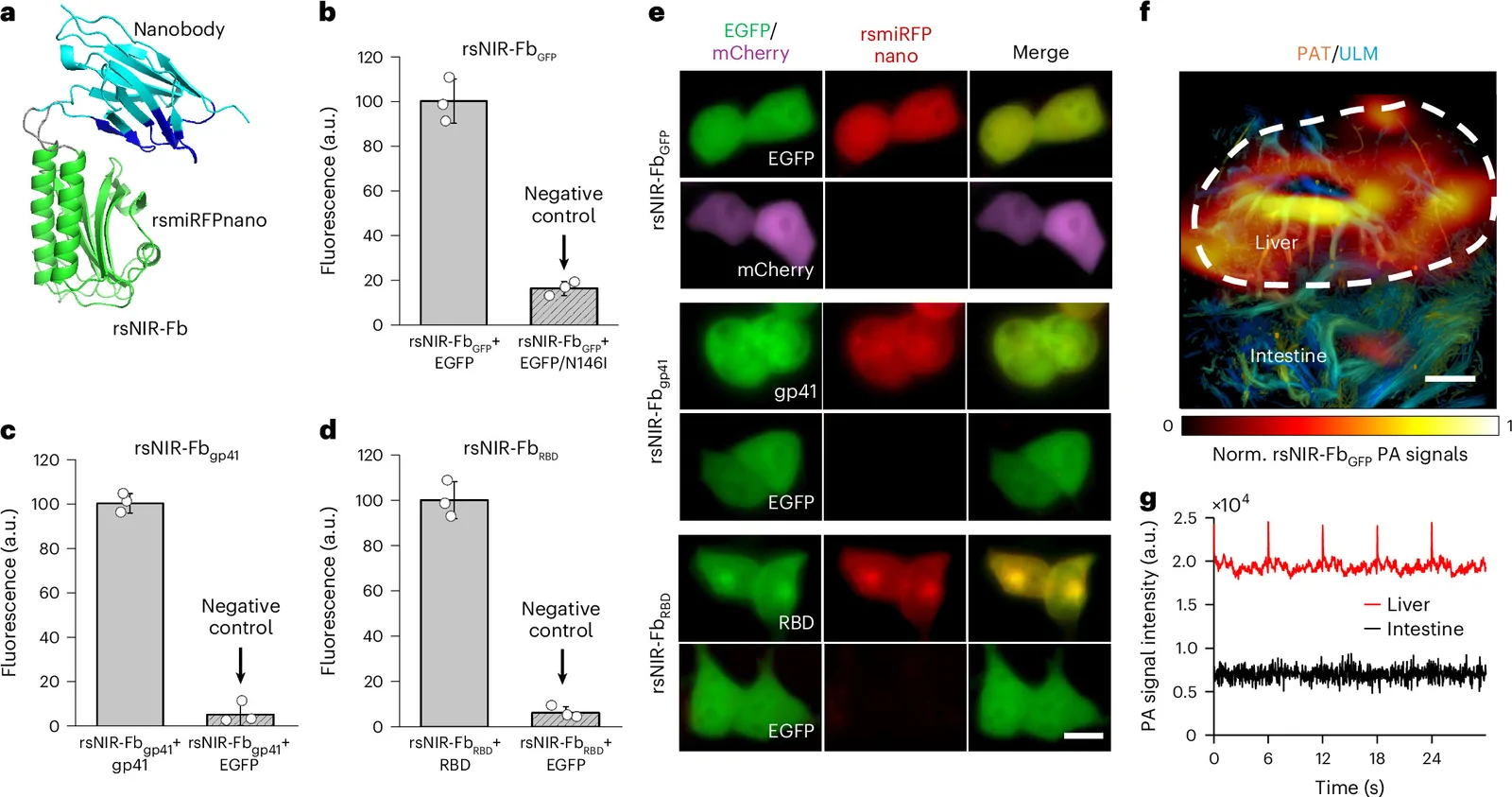
Antigen-specific PA immuno-imaging using rsNIR-Fbs. **a**, AlphaFold3 model of rsNIR-Fb_GFP_, showing rsmiRFPnano (green), the nanobody scaffold (blue), and CDRs (dark blue). **b**–**d**, Fluorescence intensity of HEK293T cells transfected with rsNIR-Fb_GFP_ (**b**), rsNIR-Fb_gp41_ (**c**) and rsNIR-Fb_RBD_ (**d**). For each rsNIR-Fb, cells were co-transfected with the cognate antigen or a nonbinding control (EGFP/N146I mutant in **b**, or EGFP in **c**,**d**). Data are presented as mean ± s.d., *n* = 3 independent transfection experiments. **e**, Representative fluorescence images of cells coexpressing each rsNIR-Fb with its cognate antigen (top) or the corresponding control protein (bottom). Fluorescence imaging was performed using excitation/emission filter sets of 685/20 nm−725/40 nm for rsmiRFPnano, 485/20 nm−525/30 nm for EGFP, and 560/25 nm−607/36 nm for mCherry. Images are representative of three independent experiments. Scale bar, 10 µm. **f**, Representative in vivo hybrid differential PAT/ULM image showing the liver vasculature resolved by ULM and rsNIR-Fb_GFP_ expression detected by differential PAT. Images are representative of *n* = 3 mice. Scale bar, 2 mm. **g**, Dynamic PA signals extracted from the liver and intestine regions shown in **f**. Source Data

To determine whether insertion of a GAF domain is structurally compatible with Nb folding and specificity, we performed AlphaFold3 modeling^44^ of rsNIR-Fb_GFP_ and compared the predicted structure with the crystal structure of Nb_GFP_ in complex with EGFP (PDB ID 3OGO) (Fig. 6a). Structural superposition showed that the rsNIR-Fb model retains the canonical VHH β-sandwich architecture observed in the experimental Nb_GFP_ structure. The global Nb fold is preserved, with minimal deviations across framework region (FR) residues and no substantial perturbation of the antigen-binding CDR surface. Structural differences are confined to the insertion and linker region, consistent with localized conformational flexibility and strain at the internal fusion site. This structural comparison of rsNIR-Fb_GFP_ and Nb_GFP_ shows that internal fusion of rsmiRFPnano induces localized structural remodeling while maintaining the Nb architecture required for antigen binding.

To demonstrate the generality of this predicted rsNIR-Fb design, the same FR3 insertion position and configuration of both linkers were further used to develop rsNIR-Fbs recognizing distinct antigens, including EGFP, HIV-1 gp41 protein and SARS-CoV-2 spike protein (Supplementary Fig. 19).

### Validation of rsNIR-Fbs in live mammalian cells and in vivo

Because rsmiRFPnano is fluorescent in its 715-nm-absorbing ground state, NIR fluorescence provided a convenient and sensitive readout to assess rsNIR-Fb stabilization upon antigen binding. To characterize the performance of rsNIR-Fbs in mammalian cells, we coexpressed them with their cognate antigens. rsNIR-Fb_GFP_ was coexpressed with either EGFP or with EGFP/N146I mutant or mCherry controls not recognized by this Nb. rsNIR-Fb_gp41_ and rsNIR-Fb_spike_ were coexpressed either with their corresponding cognate antigens fused to EGFP for visualization of co-transfection or with unfused EGFP as a control.

Flow cytometry of the live mammalian cells revealed robust rsNIR-Fb fluorescence in the presence of cognate antigens and negligible signals with nonbinding controls (Fig. 6b–d), confirming antigen specificity and antigen-dependent stabilization. Live-cell fluorescence microscopy further supported these results, showing strong intracellular NIR signals only in cells coexpressing rsNIR-Fbs and their respective antigens, whereas control conditions exhibited near-background fluorescence (Fig. 6e). Together, these results demonstrated that rsmiRFPnano insertion preserved the high antigen specificity and conditional intracellular stabilization while introducing reversible photoswitching.

To assess whether antigen-dependent stabilization can be combined with photoswitching-based PAT in vivo, we performed proof-of-concept experiments in mice using rsNIR-Fb_GFP_. EGFP was selected as a model intracellular antigen because it is a widely used marker that provides independent fluorescence readout. The experimental workflow combined liver-restricted EGFP expression induced by hydrodynamic transfection with systemic administration of adeno-associated virus (AAV)-encoded rsNIR-Fb_GFP_ via tail-vein injection (Supplementary Fig. 20). Hydrodynamic transfection resulted in predominantly hepatocyte-localized EGFP expression, whereas rsNIR-Fb_GFP_ was delivered systemically using an AAV vector. In vivo differential PAT, combined with ULM, revealed robust, spatially confined rsNIR-Fb-derived PA signals in the liver, co-registered with the hepatic microvasculature, whereas no PA signals were detected in other organs, such as the intestine control region (Fig. 6f,g). Strong PAT signals in the EGFP-expressing liver indicated the rsNIR-Fb stabilization upon binding EGFP.

To further validate the antigen-dependent activation mechanism in vivo, we performed control experiments using the same experimental workflow except that the EGFP plasmid was replaced with PBS during hydrodynamic injection (Supplementary Fig. 21). Under these conditions, mice received systemic delivery of rsNIR-Fb_GFP_ but did not express the target EGFP antigen in the liver. In contrast to the EGFP-expressing animals, virtually no differential PAT signals were detectable in vivo, and no detectable photoswitching-associated signal modulation was observed (Supplementary Fig. 22). These results support that the observed rsNIR-Fb_GFP_ signals primarily arise from antigen-dependent stabilization rather than spontaneous probe activation or nonspecific accumulation.

Collectively, these findings establish rsNIR-Fbs as antigen-dependent, photochromic probes that enable selective photoacoustic immuno-imaging. rsNIR-Fbs allow specific detection of intracellular targets even within highly vascularized organs, where strong blood-derived absorption typically limits molecular specificity. The ability to confine rsNIR-Fb-derived PA signals to antigen-expressing tissue and to spatially resolve them relative to the microvasculature demonstrates the potential of this immuno-PAT approach for complex physiological environments.

## Discussion

rsmiRFPnano represents the most compact CBCR-derived reversibly switchable probe for in vivo differential PAT. Engineered through molecular evolution of the JSC1_58120g3 CBCR domain, rsmiRFPnano combines an ultracompact size of 18.8 kDa, efficient expression in mammalian cells and reversible photoswitching between the NIR and far-red states (Fig. 1), enabling robust photoacoustic signal modulation in vivo. Notably, the molecular weight of rsmiRFPnano is threefold smaller than the most compact BphP-derived PAT probes reported to date^14–16^. This architecture provides a favorable and flexible platform for the design of genetically encoded PAT reporters.

An important finding in our study is that the strong CNR observed using rsmiRFPnano for differential PAT in whole blood, similar to that in a blood-free phantom, demonstrates that the photoswitching-derived contrast is virtually independent of the surrounding optical environment. The ability of rsmiRFPnano to maintain high detectability despite strong hemoglobin absorption highlights its exceptional photoswitching efficiency and substantial PAT sensitivity. Furthermore, the stable photochromic performance of rsmiRFPnano over multiple cycles enables consistent quantitative metrics during longitudinal imaging. These phantom-based results provide a foundation for high-sensitivity molecular readouts in optically dense, hemoglobin-rich tissues in vivo.

Application of rsmiRFPnano-based reporters in an in vivo liver inflammation model demonstrates that differential PAT can support noninvasive, longitudinal monitoring of endogenous transcriptional activity in deep tissues. Of note, this approach enables resolution of pathway-specific signaling dynamics within intact organs under conditions of strong endogenous optical absorption. The capacity to noninvasively discriminate distinct temporal activation profiles of NF-κB and AP-1 highlights the sensitivity of rsmiRFPnano-based PA readouts for capturing endogenous transcriptional responses in highly absorbing tissue environments and positions this strategy as a general platform for interrogating immune-regulatory signaling pathways and disease-associated transcriptional programs in deep tissues.

Complementing the molecular PAT readouts, the same imaging platform enabled hemodynamic assessment through the ULM modality, providing an integrated functional perspective on inflammation-induced vascular alterations (Extended Data Fig. 2). By combining pathway-specific molecular imaging with super-resolution microvascular flow measurements, this multicontrast approach offers a more comprehensive characterization of inflammatory responses than either modality alone. The observed hemodynamic changes are consistent with vascular responses commonly observed in acute inflammation, including endothelial activation, leukocyte–endothelial interactions and impaired capillary perfusion^45^, providing functional context for the molecular and cellular inflammatory processes identified by in vivo imaging.

Crucially, multiplexed spatial omics analysis provided an independent, ex vivo cellular and molecular context for interpreting the in vivo PAT observations. Spatial omics served as an orthogonal validation approach, confirming that regions identified as inflamed in vivo corresponded to well-defined inflammatory cellular states at the tissue level. This separation between noninvasive whole-organ imaging and ex vivo spatial profiling strengthens confidence in the biological specificity of the rsmiRFPnano-based reporters while preserving a clear distinction between in vivo functional imaging and downstream molecular validation. In this context, rsmiRFPnano functions not merely as a contrast agent but as a genetically encoded biosensor capable of reporting inflammation-associated pathological states in living tissue, providing a robust platform for studying inflammatory disease progression and evaluating therapeutic interventions.

Given the central role of NF-κB and AP-1 in coordinating immune responses, sustaining tissue homeostasis, and driving pathological states such as chronic inflammation and oncogenesis^25,26^, noninvasive PAT-based readouts represent a powerful platform for both basic research and preclinical applications. Beyond NF-κB and AP-1, rsmiRFPnano provides a broadly adaptable scaffold for the future development of dynamic PAT reporters targeting diverse signaling pathways. Its intrinsic reversible photoswitching allows modulation of the PA signal in real time, improving the detection sensitivity in heterogeneous tissues and supporting multiplexed imaging strategies. Because transcriptional reprogramming is central to both physiological homeostasis and disease, rsmiRFPnano-based PAT reporters could be extended to monitor processes ranging from cell activation and neuronal plasticity to oncogenic signaling and therapy response.

Using rsmiRFPnano, we developed a class of antigen-stabilized photochromic rsNIR-Fb constructs (Fig. 6). These probes accumulate selectively upon binding their cognate antigen and otherwise undergo rapid degradation^18^, thereby directly coupling probe stability and PA signal generation to the presence of the target. The in vivo validation of rsNIR-Fbs represents a critical step in translating these probes from cellular studies to whole-animal applications. Notably, even following systemic delivery and within the complex physiological environment of a living organism, rsNIR-Fb stability and PA signal generation are governed primarily by antigen presence rather than probe delivery, tissue distribution or cell-specific transcription.

The rsNIR-Fb platform is broadly applicable for PA imaging of diverse endogenous targets, including housekeeping proteins, developmental markers, intracellular signaling intermediates and viral or cancer-associated antigens, with minimal background from nonexpressing cells. In the context of immune biology, rsNIR-Fbs provide a powerful strategy for immuno-focused PAT, enabling noninvasive visualization of immune regulators and cancer-activated kinases deep within tissues. These properties position rsNIR-Fbs as a distinct class of conditional PAT probes that bridge intracellular antigen recognition and noninvasive in vivo imaging, unlocking opportunities for studying metabolic processes and disease mechanisms in tissues inaccessible by purely optical imaging modalities.

Further improving BV-binding efficiency and increasing ON–OFF photoswitching contrast should enhance the CNR of rsmiRFPnano and related probes in differential PAT, particularly at low probe abundance or BV availability. Extending rsNIR-Fbs beyond the hepatic EGFP model target to endogenous targets across diverse tissues under physiological and pathological conditions will require establishing detection limits and validating antigen-dependent stabilization.

In conclusion, rsmiRFPnano provides a versatile genetically encoded ultracompact probe for differential PAT, derived from a single CBCR domain. It substantially expands the toolkit for deep-tissue in vivo molecular imaging. The rsmiRFPnano-based reporters and antigen-dependent probes introduced here provide sensitive and specific strategies for monitoring dynamic signaling pathways and intracellular targets in vivo, with broad potential for basic biological and biomedical research.

## Methods

### Ethical statement

All procedures with animals were performed in an AAALAC-approved facility and received ethical approval from the Institutional Animal Care and Use Committee of Duke University (protocol A203-22-12 and 60681).

### Housing and husbandry

Mice were maintained on a 12-h light–dark cycle at an ambient temperature of 20–24 °C and standard humidity of ~40–55%, with ad libitum access to food and water. Same-sex littermates were housed together in cages with chopped corn cob bedding (2–5 mice per cage). Environmental enrichment included pieces of compressed cotton nestlets and paper huts.

### Mutagenesis and directed molecular evolution

Synthetic genes encoding CBCR GAF domains were obtained from GenScript, with sequences codon-optimized for mammalian expression using the OptimumGene algorithm. Optimization accounted for codon usage bias, GC content, CpG frequency and predicted mRNA secondary structure. For bacterial expression, genes were subcloned into the pDRESS plasmid (Addgene, 189755) using NcoI/BamHI restriction sites. To provide BV, cells were co-transformed with a pBAD plasmid encoding the *Bradyrhizobium* ORS278 heme oxygenase (*hmuO*). Protein expression was carried out in *E.* *coli* TOP10 cells (Invitrogen).

Mutagenesis was performed using an overlap-extension PCR strategy to introduce targeted substitutions at multiple sites. The resulting libraries were transformed into BV-producing TOP10 cells and cultured overnight at 37 °C in LB medium containing ampicillin and kanamycin. For induction, 0.02% rhamnose was added to the medium, and bacterial colonies were subsequently grown on selective agar plates containing the same inducer and antibiotics.

Colonies were screened for NIR fluorescence using a Leica M205 fluorescence stereomicroscope (700/20 nm excitation, 730 nm long-pass emission filters; Chroma) equipped with a Tucsen CCD camera. Approximately 20 clones showing the highest brightness were selected for further evaluation in transiently transfected HeLa cells.

### Bacterial plasmids and protein expression

Genes encoding rsmiRFPnano or wild-type JSC1_58120g3 were cloned with an N-terminal polyhistidine tag and expressed in *E.* *coli* TOP10 cells. Protein expression was induced with 0.02% rhamnose, followed by incubation for 5 h at 37 °C and an additional 20 h at 22 °C in LB medium supplemented with ampicillin and kanamycin. Proteins were purified using Ni–NTA agarose (QIAGEN), eluted in PBS containing 100 mM EDTA, and desalted on PD-10 columns (GE Healthcare).

Absorbance spectra were recorded with a Hitachi U-2000 spectrophotometer. Extinction coefficients were determined from the ratio of the Q-band to the Soret band, assuming a reference value of 39,900 M⁻^1^ cm⁻^1^ for the Soret band^46^. To record spectra of the far-red-absorbing photoproduct, proteins were illuminated with a custom 740 nm LED positioned orthogonally to the spectrophotometer optical path. Reversion to the NIR-absorbing state was achieved with 660 nm illumination. In both cases, spectra were acquired only after illumination was completed to prevent interference from the excitation source.

Fluorescence measurements were performed using a Cary Eclipse spectrofluorometer (Agilent Technologies). For comparison of molecular brightness, protein solutions were normalized to equal absorbance at the Soret band (385 nm) to ensure identical chromophore content. Fluorescence emission spectra were then recorded under identical conditions, and integrated intensities were compared between rsmiRFPnano and its parental protein. Data processing and statistical analyses were conducted using OriginPro 2021b (OriginLab) and Microsoft Excel v.15.36.

### Quantum yield determination

Fluorescence quantum yield (QY) of rsmiRFPnano in the ground state was determined relative to miRFP718nano protein, for which the QY has been previously reported to be 5.6%. Protein samples were purified using Ni–NTA affinity chromatography and eluted in 100 mM EDTA in PBS, pH 7.6.

Serial dilutions of protein preparations were prepared in PBS to obtain a range of concentrations with absorbance values at the Q-band maximum not exceeding 0.1, to minimize inner filter effects. Before measurements, protein samples were kept in complete darkness at room temperature for 1 h to ensure complete equilibration to the ground state. All measurements were performed under minimal light exposure to avoid photoconversion to the photoswitched state.

Absorption spectra were recorded using a U-3010 spectrophotometer (Hitachi) with a 1-cm pathlength quartz cuvette (Starna Cells, sub-micro cuvette of 100 µl). Light-scattering contributions were subtracted from the absorption spectra using OriginPro.

Fluorescence emission spectra were recorded using a FluoroMax-3 spectrofluorometer (Jobin Yvon, Horiba) with 1.5 ml PMMA cuvettes (BRAND, 759085D). Emission spectra were processed and integrated using OriginPro to obtain total fluorescence intensity.

For each protein, the integrated emission intensity was plotted against the corresponding absorbance at 700 nm, and linear regression was applied to determine the slope (*k*) of the resulting dependence. The QY of rsmiRFPnano was calculated from the ratio of slopes according to:

$${\mathrm{QY}}_{\mathrm{rsmiRFPnano}}={\mathrm{QY}}_{\mathrm{miRFP}718{\mathrm{nano}}}\times \frac{{k}_{\mathrm{rsmiRFPnano}}}{{k}_{\mathrm{miRFP}718{\mathrm{nano}}}}$$

All measurements and data processing steps were performed under identical conditions for the sample and the reference protein.

### pH measurements

rsmiRFPnano protein was purified using Ni–NTA affinity chromatography and eluted in PBS (pH 7.6) supplemented with 100 mM EDTA. The protein was further concentrated using Amicon Ultra-4 centrifugal filter units (10 kDa molecular weight cutoff) to a final concentration of approximately 13.4 mg ml^−1^.

The concentrated protein was diluted 100-fold into a series of pH standard buffers with pH values of 2.14, 3.17, 4.07, 5.16, 6.25, 7.08, 7.95, 8.80, 9.47 and 9.88.

Before measurements, samples were kept in complete darkness at room temperature (RT) for 1 h to ensure equilibration to the ground state. Absorption spectra were recorded using a U-3010 spectrophotometer (Hitachi) with a 1-cm pathlength quartz cuvette (Starna Cells, sub-micro cuvette of 100 µl). Measurements were performed under minimal light exposure to prevent unintended photoconversion.

For induction of the photoswitched state, samples were illuminated with a 740/15 nm LED (6.5 mW cm^−2^) powered by a QW-MS3010D DC power supply for 10 min at RT. Following illumination, absorption spectra were recorded again under minimal light exposure.

Light-scattering contributions were subtracted from all absorption spectra using OriginPro. To correct for background signal arising from denatured protein and covalently bound biliverdin, the absorption spectrum obtained at pH 12.3 (where the protein is fully denatured) was subtracted from all other spectra.

The pH dependence of absorbance was analyzed at the maxima of the Q-bands corresponding to the ground and photoswitched states. The resulting data were plotted as a function of pH and fitted using a sigmoidal function describing a single protonation equilibrium:

$$F={F}_{\min }+\frac{{F}_{\max }-{F}_{\min }}{1+{10}^{({\rm{p}}{K}_{a}-{\rm{pH}})}}$$

where, ${F}_{\min }$ is the baseline absorbance at low pH, ${F}_{\max }$ is the maximum at high pH, and ${\rm{p}}{K}_{a}$ is the apparent dissociation constant. Nonlinear fitting was performed using OriginPro, and ${\rm{p}}{K}_{a}$ values were extracted for both the ground and photoswitched states.

### Native PAGE

The oligomeric state of the proteins was assessed by native PAGE. Purified rsmiRFPnano and miRFP713 proteins were obtained by Ni–NTA affinity chromatography and eluted in PBS (pH 7.6) supplemented with 100 mM EDTA and 10 mM β-mercaptoethanol. Protein concentrations were determined from absorbance at 280 nm using an extinction coefficient of 26,630 M⁻^1^ cm⁻^1^, calculated from the amino acid sequence.

Glycerol was added to the protein samples to a final concentration of 12.5%. Different amounts of protein were loaded onto Mini-PROTEAN TGX precast gels (Bio-Rad, 4561094). Electrophoresis was performed in 1× Tris/glycine running buffer (Bio-Rad, 1610734) at 100 V for 100 min.

Fluorescent protein bands were visualized directly in the gel using a Newton FT-900 imaging system (Viber) with excitation at 680 nm and a 750 nm long-pass emission filter. Corresponding bright-field (daylight) images of the gels were acquired and used to correlate fluorescent signals with total protein band positions.

### Construction of mammalian plasmids

Genes encoding rsmiRFPnano or its variants were cloned into the pDRESS vector (Addgene, 189755) using NcoI and BamHI restriction sites. To generate the pTRE-rsmiRFPnano vector, the EGFP gene in the pTRE-EGFP vector (Addgene, 89871) was replaced with the rsmiRFPnano gene. For engineering of nanobody (Nb) fusions, rsmiRFPnano was inserted internally into Nb scaffolds by overlap PCR, and the resulting constructs were subcloned into pcDNA vectors at the KpnI/EcoRI sites. The anti-GFP Nb was amplified from the pcDNA3.1-NSlmb-vhhGFP4 plasmid (Addgene, 35579), whereas Nbs specific for HIV-1 gp41 and the SARS-CoV-2 spike protein were synthesized de novo (GenScript).

To prepare antigen expression constructs, the gene encoding the SARS-CoV-2 spike receptor-binding domain (RBD; residues 333–529) was amplified from pDONR223-SARS-CoV-2 S (Addgene, 149329), and the gp41 gene was amplified from pLAI-Env (Addgene, 133996). Both antigens were fused to mEGFP (Addgene, 62237) by splicing-by-overlap-extension PCR and cloned into a pcDNA3.1 vector.

For transcriptional reporters, the rsmiRFPnano-based NF-κB reporter was generated by replacing the miRFP718nano gene in a previously described inflammation reporter^19^ plasmid with rsmiRFPnano. The AP-1 reporter was derived from this construct by substituting the NF-κB response elements (gggaatttccggggactttccgggaatttccggggactttccgggaatttcc) with six tandem AP-1 elements (tgagtcagtgactcagtgagtcagtgactcagtgagtcagtgactcag).

To construct pAAV-rsNIR-Fb-GFP plasmid, the rsNIR-Fb_GFP_ gene was inserted into an AAV-based vector with a CAG promoter^47^ using KpnI/EcoRI sites.

### Mammalian plasmids, cell culture and transfection

HeLa (CRM-CCL-2) and HEK293T (CRL-3216) cells (ATCC) were maintained in DMEM with 10% fetal bovine serum, 2 mM L-glutamine and 0.5% penicillin–streptomycin (all Thermo Fisher Scientific) at 37 °C in 5% CO_2_. For live-cell microscopy, cells were seeded in 35-mm glass-bottom dishes (Greiner Bio-One). Transient transfections used polyethyleneimine (PEI) following standard protocols^48^.

To compare molecular brightness, HEK293T cells were transiently co-transfected with plasmids encoding rsmiRFPnano or wild-type JSC1_58120g3 and pEGFP as a transfection marker, and fluorescence was quantified 48 h later by flow cytometry.

For reporter assays, HEK293T cells were transfected with rsmiRFPnano-based NF-κB or AP-1 reporters plus pEGFP (1:10) to mark transfected cells, stimulated 48 h later with TNF (NF-κB) or PMA (AP-1) at the indicated concentrations, and analyzed by flow cytometry and fluorescence microscopy under identical conditions.

To assess rsNIR-Fbs, HEK293T cells were co-transfected with individual rsNIR-Fbs and either the cognate antigen or a control protein (1:1), and NIR fluorescence was analyzed 72 h later by flow cytometry and fluorescence microscopy.

### Mammalian cell fluorescence imaging

Live cells were imaged with an Olympus IX81 inverted epifluorescence microscope equipped with a Xenon lamp (Lambda LS, Sutter). Images were acquired using an ORCA-Flash4.0 V3 camera (Hamamatsu) and a ×60 1.35 NA oil-immersion objective lens (UPlanSApo, Olympus). During imaging, cells were maintained in a cell imaging solution (Life Technologies–Invitrogen) at 37 °C. The microscope was operated with SlideBook v.6.0.8 software (Intelligent Imaging Innovations).

For rsmiRFPnano, a 685/20 nm excitation filter and a 725/40 nm emission filter (Chroma) were used. EGFP was imaged using a 485/20 nm excitation and a 525/30 nm emission filter, and mCherry was imaged with a 560/25 nm excitation and a 607/36 nm emission filter. Image analysis was performed with SlideBook v.6.0.8 and Fiji v.1.50b software.

### Flow cytometry

Flow cytometry was performed on a BD Accuri C6 flow cytometer equipped with 488 nm and 640 nm lasers and corresponding emission filters. rsmiRFPnano and JSC1_58120g3 fluorescence were detected using a 670 nm long-pass filter. EGFP was excited with the 488 nm laser and detected with a 510/15 nm filter. For each sample, 20,000–50,000 events were collected. Gating was performed as shown in Supplementary Fig. 23. Data were processed using FlowJo v.7.6.2 software.

### Intracellular stability of rsmiRFPnano in mammalian cells

To determine intracellular stability of rsmiRFPnano, HEK293T cells transiently expressing rsmiRFPnano were treated with cycloheximide (50 μg ml⁻^1^) to inhibit protein synthesis. At 0, 2, 4, 6, 8 and 24 h after treatment, cells were collected and analyzed by flow cytometry. Background fluorescence from non-transfected cells was subtracted, and fluorescence values were normalized to the value at the 0 h time point. The decay kinetics were fitted in Origin software using a one-phase exponential decay model with the baseline constrained to zero. The apparent fluorescence half-life was calculated from the fitted decay constant.

### Apparent maturation kinetics of rsmiRFPnano in mammalian cells

To evaluate the maturation kinetics of rsmiRFPnano in mammalian cells, HEK293T cells were co-transfected with pTRE-rsmiRFPnano and pTRE-EGFP plasmids together with the Tet-On plasmid at a ratio of 6:1:3. Expression was induced by addition of doxycycline (1 μg ml⁻^1^) at time 0. Cells were collected at 6, 12, 24, 36, 48, 60 and 72 h after induction and analyzed by flow cytometry. The fluorescence accumulation kinetics were fitted in Origin software using a sigmoidal function, and the apparent half-time of fluorescence accumulation was calculated from the fitted curve.

### Hybrid 3D photoacoustic and ultrasound localization microscopy system

The hybrid system comprises a programmable ultrasound scanner (Vantage 256, Verasonics), multiple pulsed lasers for PAT excitation, and a customized two-dimensional (2D) semispherical 256-element ultrasound transducer array (Imasonics) for both PAT and US imaging^12^. The array has a central opening for inserting an optical fiber bundle for PAT excitation. The array has a center frequency of 4 MHz, with a −6 dB bandwidth of ~45% in the transmit/receive mode, fine-tuned to maximize the receiving bandwidth. It has a curvature radius of 40 mm, a total aperture of 57 mm, and a well-sampled three-dimensional (3D) field of view (the focal zone) of 8 × 8 × 8 mm^3^. While imaging, the array faced up inside a temperature-regulated water bath for acoustic coupling, and the animal was mounted on an optically and acoustically transparent holder at the focal zone, which was raster-scanned by a 2D motorized translation stage for an expanded 3D field of view. The system has two imaging modes, differential PAT and ULM, described in detail below.

### 3D differential PAT

To photoswitch and image rsmiRFPnano, we combined two CW semiconductor lasers at 635 nm and an optical parametric oscillator (OPO) laser (Surelite, Continuum) pumped by a 30 Hz Nd:YAG laser. The 725 nm OPO light was used simultaneously for PA imaging and for switching rsmiRFPnano OFF, while the 635 nm CW light switched it ON. Both beams were combined by a dichroic mirror and delivered through the optical fiber bundle (Supplementary Fig. 6). The beam diameter on the animal surface was ~1 cm, and the maximum fluence on the skin was ~5 mJ cm^−2^, below the American National Standards Institute laser safety limit. At each scanning location, five photoswitching cycles were performed, each comprising 6 s switching ON by the 635 nm CW light and 6 s switching OFF/photoacoustic imaging by the 725 nm pulsed light, taking ~1 min per location. During imaging, animals were securely mounted in a comfortable prone position under continuous isoflurane anesthesia to minimize bulk motion. Differential PAT images were generated by subtracting the OFF-state from the ON-state images and averaging across repeated photoswitching cycles, which reduced motion-related fluctuations and improved the robustness of the differential signals.

### 3D ULM

The 3D US B-mode imaging was performed by using a synthetic aperture method^29^, and in-phase/quadrature (IQ) volumes were reconstructed with an isotropic voxel size of 60 µm. To mitigate motion artifacts due to animal breathing, 2D cross-correlation was computed on the lateral maximum-intensity-projection (MIP) images using the averaged MIP frame as reference, and frames below a 99% correlation threshold (~10% of total frames) were removed. The ULM workflow then reconstructed the vasculature in four steps (Supplementary Fig. 15).

(1) Spatial-temporal singular value decomposition (SVD) within each block of 600 B-mode volumes removed clutter and extracted microbubble signals, discarding the 150 largest singular values per block; (2) power Doppler (PD) volumes were formed by integrating the SVD-filtered IQ data, with directional and bandpass filtering (20–107 Hz) to facilitate microbubble separation; (3) a radial-symmetry-based algorithm localized microbubbles at sub-diffraction resolution; and (4) a 3D particle-tracking algorithm tracked microbubbles across consecutive volumes, using a maximum pairing/linking search radius of 10 voxels (corresponding to a maximum bubble velocity of ~130 mm s^−1^), a maximum gap-closing window of 1 (disabling merging of discontinuous tracks), a linear-motion Kalman filter model with default initial state, and a maximum linking angle of 45°. The accumulated microbubble trajectories generated the ULM density map, and a microbubble flow-velocity map was computed from the trajectories to provide blood-flow information.

### Wide-field fluorescence imaging for validating photoacoustic imaging

A laboratory-made wide-field fluorescence system used a high-sensitivity InGaAs camera with a broad detection spectrum from 600 nm to 1.7 μm (Ninox 640 II, Raptor Photonics; 15-μm pixels, maximum frame rate 120 Hz), equipped with an F/1.8 objective lens (RPL-OESWIRECON14mmx1.8A, Raptor Photonics). Excitation was provided by a 680 nm laser diode (M660L4, Thorlabs) with a 680/10 nm filter (FB680/10, Thorlabs) at a surface excitation intensity of ~50 mW cm^−2^, and emission was collected through a 750/40 nm filter (FB750-40, Thorlabs). Unless otherwise stated, the camera exposure time was 20 ms for all images.

### AAV preparation and characterization

Recombinant AAV vectors were produced by triple-plasmid transfection: HEK293T cells at ~70% confluency were co-transfected with pAAV2/8 (Addgene, 112864), pHelper (Addgene, 112867) and the transgene plasmid pAAV-rsNIR-Fb-GFP at a 1:1:1 molar ratio using polyethyleneimine (PEI). At 72 h post-transfection, cells were collected and lysed, and viral particles were purified with the AAVpro Purification kit (Takara) per the manufacturer’s instructions.

Before titration, preparations were treated with DNase to remove unpackaged DNA, and viral genome titers were quantified as DNase-resistant genome copies by SYBR Green quantitative PCR (qPCR) on a QuantStudio Real-Time PCR System (Applied Biosystems), using primers specific for rsmiRFPnano (forward 5′-GACCTGGAGAGCAAGTTCAG-3′; reverse 5′-GTGATGAAGTCGCCGAAGTA-3′).

### Mouse procedures and hydrodynamic liver transfection

All animal experiments used female *Blvra*^−^^/^^−^ mice (6–8 weeks old) on a C57BL/6 (B6 albino) background. The *Blvra*^−/−^ strain was a gift from K. Terai and M. Matsuda, obtained via the Japanese Collection of Research Bioresources (JCRB) Laboratory Animal Resource Bank (NIBIOHN; Tokyo, Japan; animal ID nbio-370); it was generated by CRISPR/Cas9 targeting of the murine *Blvra* gene (NM_026678.4) and backcrossed to B6 albino mice^12^. Hydrodynamic liver transfection followed an established protocol. Mice were anesthetized with isoflurane (1–1.5% v/v) and given a rapid tail-vein injection of plasmids encoding the NF-κB reporter, the AP-1 reporter (both based on rsmiRFPnano) or a GFP control (Addgene, 129020). The injection volume was 8% of body weight (1.6 ml of 20 µg ml⁻^1^ plasmid solution within 10 s for a typical 20-g mouse). Mice were then allowed to recover under standard housing. At 72 h post-transfection, mice received an intraperitoneal injection of LPS in PBS (2 mg kg⁻^1^, 100 µl), and PAT and fluorescence imaging began 12 h later and continued for 5 days. Negative controls received an equal volume of PBS instead of LPS, with all other procedures identical.

To evaluate antigen-dependent stabilization and photoswitching-dependent PAT of rsNIR-Fb_GFP_ in vivo, an EGFP-encoding plasmid was first delivered by hydrodynamic transfection to induce liver-restricted EGFP expression; 24 h later, an AAV vector encoding rsNIR-Fb_GFP_ was administered by tail-vein injection, and in vivo imaging was performed 4 days after AAV delivery. Negative controls received PBS instead of the EGFP-encoding plasmid during the hydrodynamic injection, with all other procedures identical.

### CODEX multiplexed imaging

Liver tissues were embedded in OCT, frozen and cryosectioned at 7 µm (Epredia HM525 NX). Sections were stained with validated CODEX antibody panels and imaged by cyclic stripping, annealing and imaging of fluorescently labeled oligonucleotides complementary to antibody-conjugated barcodes^30,31^. Raw data were processed in Akoya PhenoCycler Fusion v.2.2.0 (stitching, drift compensation, deconvolution and cycle concatenation); markers with untenable patterns or low signal-to-noise were excluded, and images were visualized in QuPath (https://qupath.github.io/).

### CODEX single-cell segmentation

Single cells were segmented and single-cell protein expression was extracted using the SPACEc package^49^ (https://github.com/yuqiyuqitan/SPACEc/tree/master), which incorporates the deep-learning-based Mesmer segmentation method.

### CODEX cell-type analysis

Cells were classified into types and states across time points from marker expression. DAPI-positive cells of appropriate size were gated, clustering markers were *z*-normalized, and cells were over-clustered by Leiden clustering (scanpy). Clusters were annotated by mean protein expression and spatial location; impure clusters were split or re-clustered by *k*-means (scikit-learn) with reference back to the original fluorescent images.

### Statistics and reproducibility

No statistical method was used to predetermine sample size; sample sizes were based on our experience with similar experiments. Unless otherwise noted in the figure legends, at least three independent experiments were performed for in vitro and cellular assays, ≥30,000 cells were recorded per flow-cytometry sample, and animal experiments used three mice per condition, except the CODEX profiling (one mouse per time point). No data were excluded. Mice were randomized between experiments, and within each experiment all animals were age- and sex-matched littermates to reduce confounding variability. Investigators were not blinded, as the analyses relied on quantitative, operator-independent readouts. Data distribution was assumed to be normal but not formally tested. Significance was assessed by paired two-tailed *t*-tests (indicated in the figure legends) in GraphPad Prism v.10.4.0. Data are mean ± s.d.; exact *P* values and sample sizes are given in the respective legends, where *n* denotes independent biological replicates (animals or transfection experiments). Unless otherwise noted, representative images were reproduced independently three times with similar results.

### Reporting summary

Further information on research design is available in the Nature Portfolio Reporting Summary linked to this article.

## Online content

Any methods, additional references, Nature Portfolio reporting summaries, source data, extended data, supplementary information, acknowledgements, peer review information; details of author contributions and competing interests; and statements of data and code availability are available at https://doi.org/10.1038/s41592-026-03255-y.

## Supplementary information

Supplemental Information Supplementary Figs. 1–23 and Tables 1 and 2.

Reporting Summary

Supplementary Data Source data for Supplementary Figs. 1a–d, 2–4, 7, 8 and 22b and Supplementary Table 1.

## Source data

Source Data for Main Figures Statistical source data for Figs. 1c–e, 2b,d, 3c,f, 4b,c and 6b–d,g.

Source Data for Extended Data Figures Statistical source data for Extended Data Figs. 1b and 2b,c,e,f.

## Data Availability

All data supporting the findings of this study are available within the paper and Supplementary Information. The plasmids constructed in this study are available at the Addgene repository (plasmid IDs 259781, 259783, 259785, 259810 and 259811). The nucleotide sequences of rsmiRFPnano have been deposited in GenBank under accession number PX270336. Source data are provided with this paper.

## Code availability

The image processing codes used in this study are available at Duke Photoacoustic Imaging Laboratories GitLab page at https://gitlab.oit.duke.edu/pilab/rsmiRFPnano.
