# Supplementary material for: Near-infrared photochromic miniprotein enables photoacoustic molecular imaging of acute inflammation and intracellular antigens
Source: Nat Methods. 2026 Oct 13;23(11):2274–85. doi: 10.1038/s41592-026-03255-y (PMC13736489; doi:10.1038/s41592-026-03255-y)
Supplement: Supplementary file 1 [file 41592_2026_3255_MOESM1_ESM.pdf]

nature methods

Supplementary information

https://doi.org/10.1038/s41592-026-03255-y

Near-infrared photochromic miniprotein enables photoacoustic molecular imaging of acute inflammation and intracellular antigens

In the format provided by the
authors and unedited

Supplemental Information

Table of Contents

Supplementary Figures:

Supplementary Fig. 1. Molecular engineering of rsmiRFPnano.
Supplementary Fig. 2. Absorbance spectra of wild-type JSC1_58120g3.
Supplementary Fig. 3. Fluorescence spectra of wild-type JSC1_58120g3.
Supplementary Fig. 4. Determination of pKa values for the ground and photoswitched states of rsmiRFPnano.
Supplementary Fig. 5. Native PAGE analysis of rsmiRFPnano.
Supplementary Fig. 6. Schematic of the differential PAT system.
Supplementary Fig. 7. Time course of doxycycline-induced rsmiRFPnano and EGFP expression.
Supplementary Fig. 8. rsmiRFPnano stability in HEK293T cells.
Supplementary Fig. 9. LPS-induced liver inflammation mouse model.
Supplementary Fig. 10. Photoacoustic imaging of rsmiRFPnano-based NF-κB reporters in mice upon LPS treatment.
Supplementary Fig. 11. Photoacoustic imaging of rsmiRFPnano-based AP-1 reporters in mice upon LPS treatment.
Supplementary Fig. 12. PBS-treated control mouse model.
Supplementary Fig. 13. Photoacoustic imaging of rsmiRFPnano-based NF-κB reporters in mice following PBS treatment.
Supplementary Fig. 14. Photoacoustic imaging of rsmiRFPnano-based AP-1 reporters in mice following PBS treatment.
Supplementary Fig. 15. 3D-ULM processing workflow.
Supplementary Fig. 16. Schematic overview of the CODEX multiplexed imaging and analysis workflow.
Supplementary Fig. 17. Composition of the 57-marker CODEX antibody panel used for cell type and functional state profiling.
Supplementary Fig. 18. Spatial co-registration of MALDI-IMS and CODEX imaging with cell neighborhood analysis.
Supplementary Fig. 19. Amino acid sequences of rsNIR-Fbs specific for EGFP, HIV-1 gp41 protein, and the SARS-CoV-2 spike RBD domain.
Supplementary Fig. 20. Schematic of the in vivo experimental workflow for antigen-dependent rsNIR-Fb activation in the mouse liver.
Supplementary Fig. 21. Schematic of the negative-control workflow for in vivo rsNIR-Fb imaging in the mouse liver.
Supplementary Fig. 22. Negative control for antigen-dependent rsNIR-FbGFP activation in vivo.
Supplementary Fig. 23. Exemplifying gating strategy of flow cytometry analysis.

Supplementary Tables:

Supplementary Table 1. Comparison of single-domain cyanobacteriochrome (CBCR)-derived photoswitchable photoacoustic protein rsmiRFPnano and constitutively fluorescent protein miRFP718nano.
Supplementary Table 2. Quantitative photoacoustic performance metrics of rsmiRFPnano in phosphate-buffered saline (PBS) and whole blood.

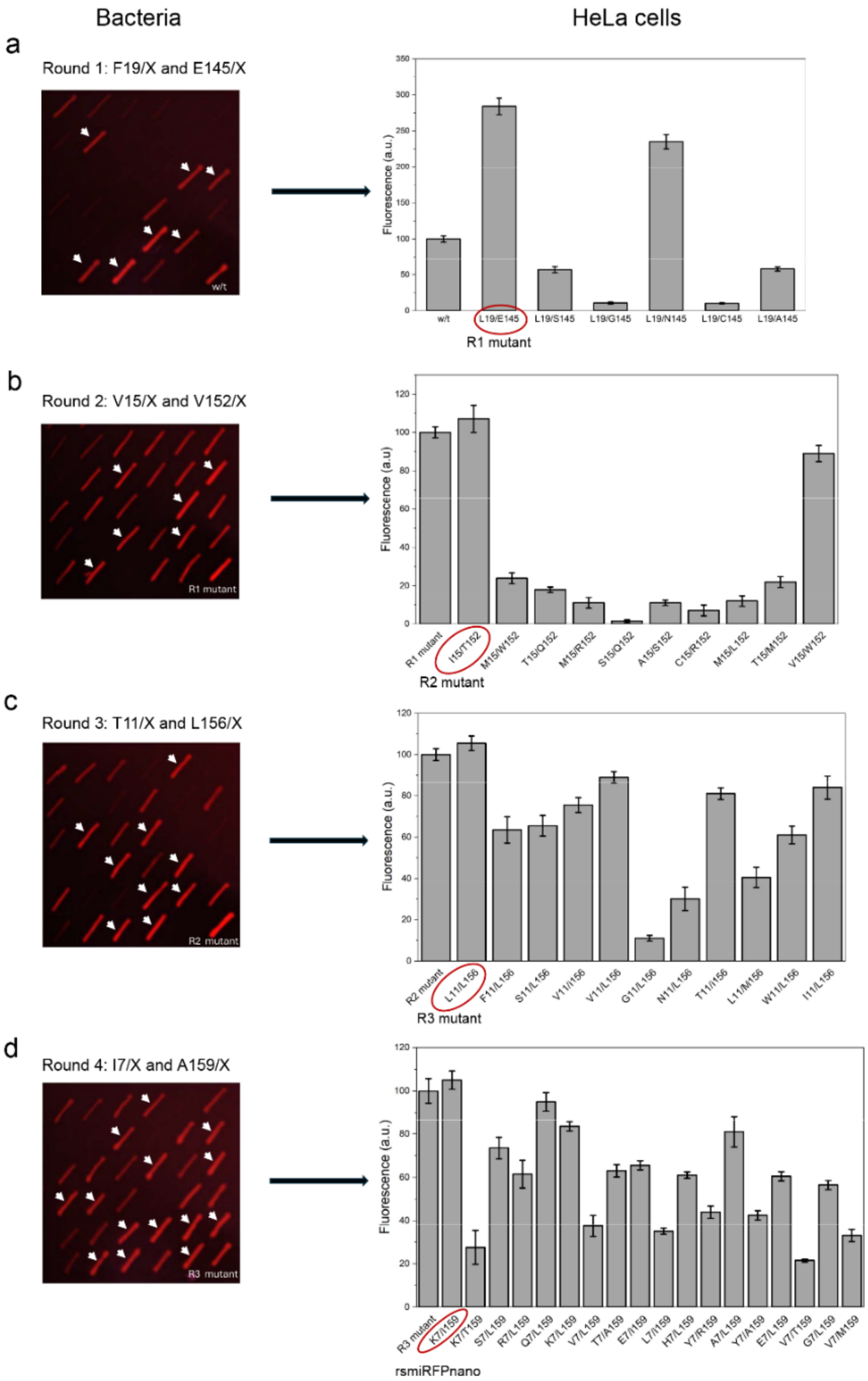

Supplementary Fig. 1. Molecular engineering of rsmiRFPnano. (a–d) Iterative rounds of simultaneous saturation mutagenesis targeting residue pairs F19 and E145 (a), V15 and V152 (b), T11 and L156 (c), and I7 and A159 (d). The best-performing clone from each round was used as a template for subsequent mutagenesis. For each round, representative bacterial plates showing obtained clones are presented (left), with selected variants indicated by asterisks. Corresponding cellular brightness of selected clones was evaluated in transiently transfected HeLa cells by flow cytometry (right). The brightness of the template clone was set to 100%. Fluorescence intensity was measured 48 h after transfection. Residues at the mutated positions and the selected variants are indicated. Data are shown as mean ± s.d. from n = 3 independent transfection experiments.

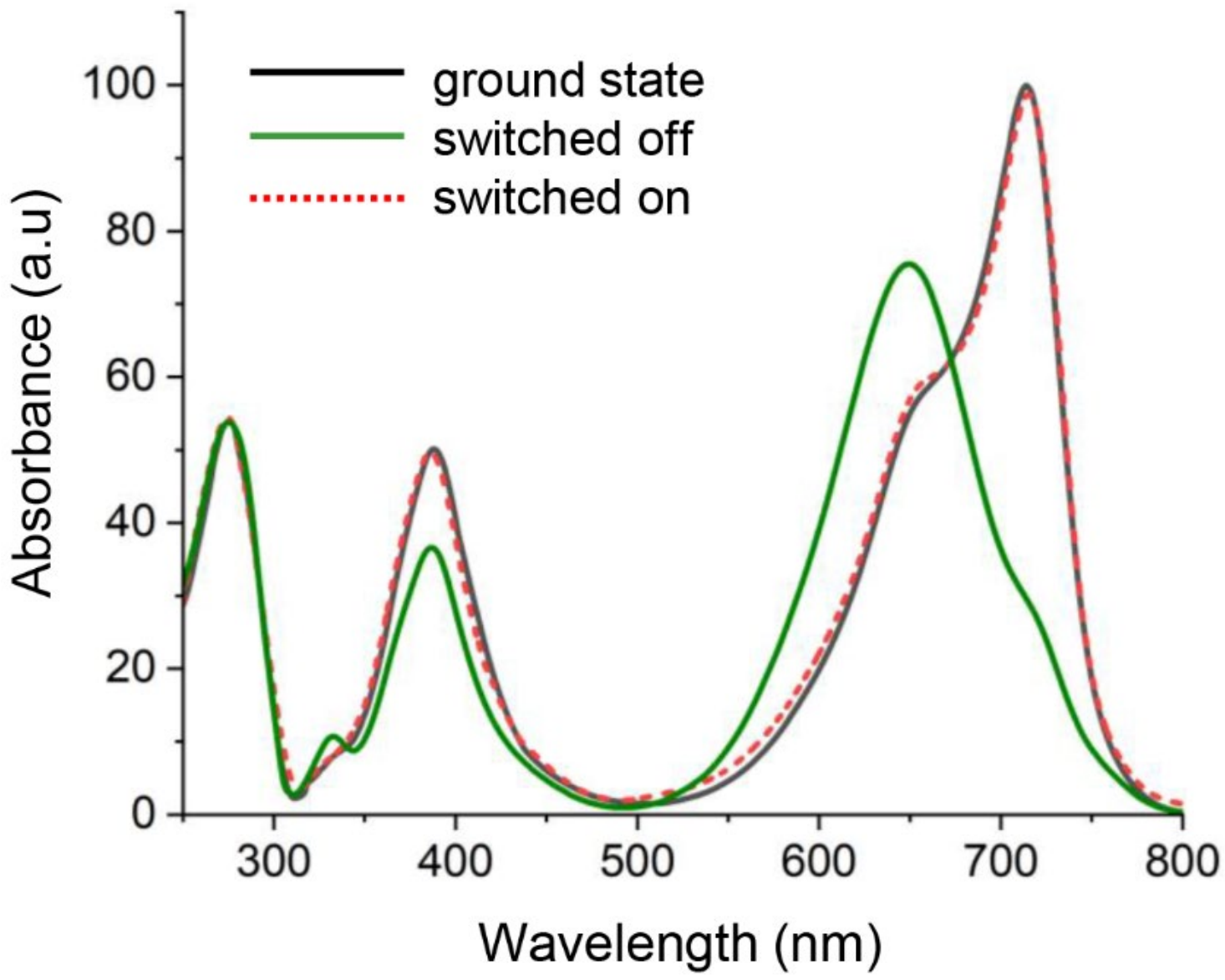

Supplementary Fig. 2. Absorbance spectra of wild-type JSC1_58120g3. The spectra in the ground NIR-absorbing state (black line), after illumination with 740 nm light (green line; switched-OFF far-red-absorbing state), and after subsequent illumination with 640 nm light (red line; switched-ON NIR-absorbing state) are shown.

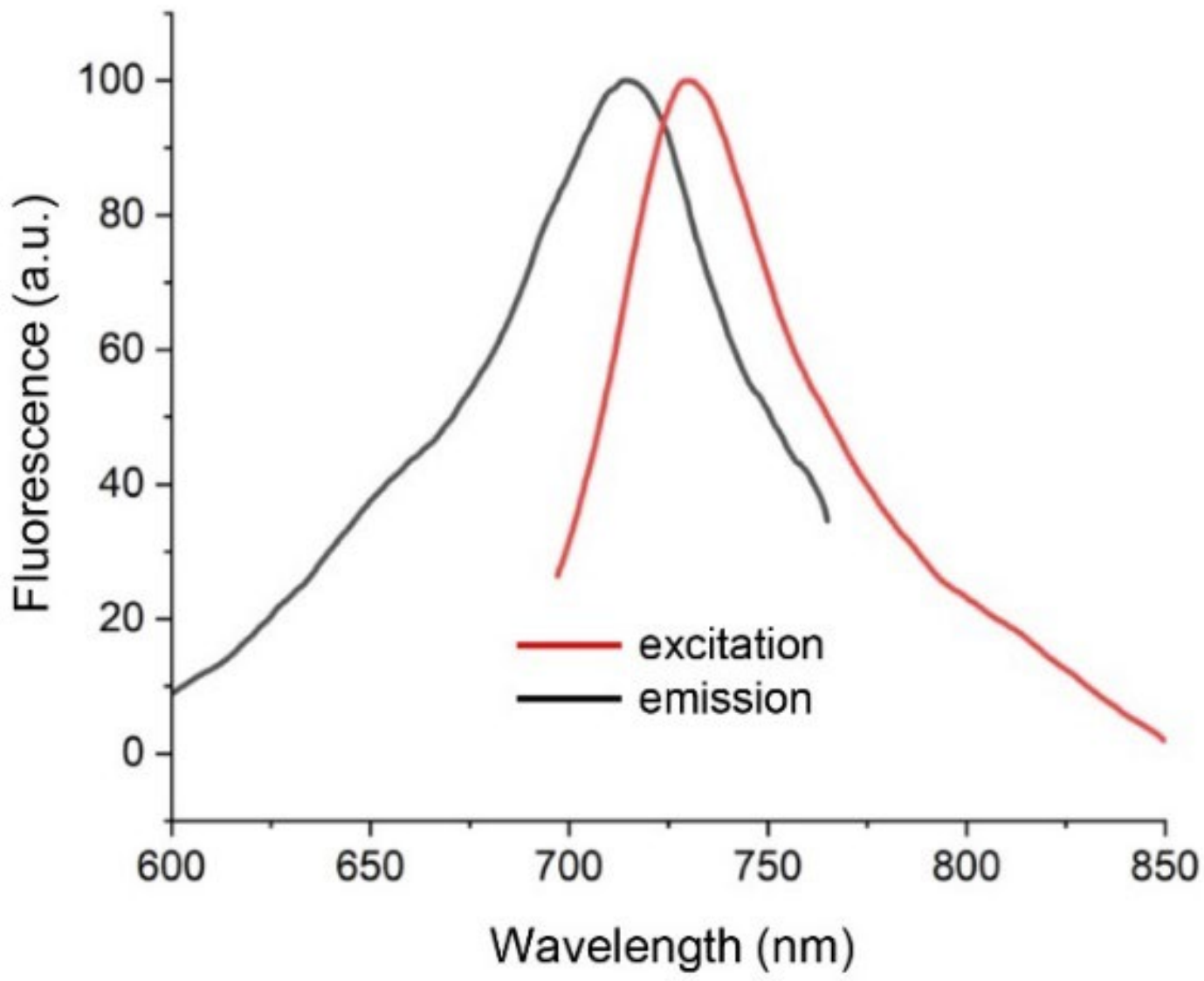

Supplementary Fig. 3. Fluorescence spectra of wild-type JSC1_58120g3. Normalized emission spectrum (black curve) was recorded upon excitation at 680 nm, and excitation spectrum (red curve) was recorded with emission monitored at 770 nm.

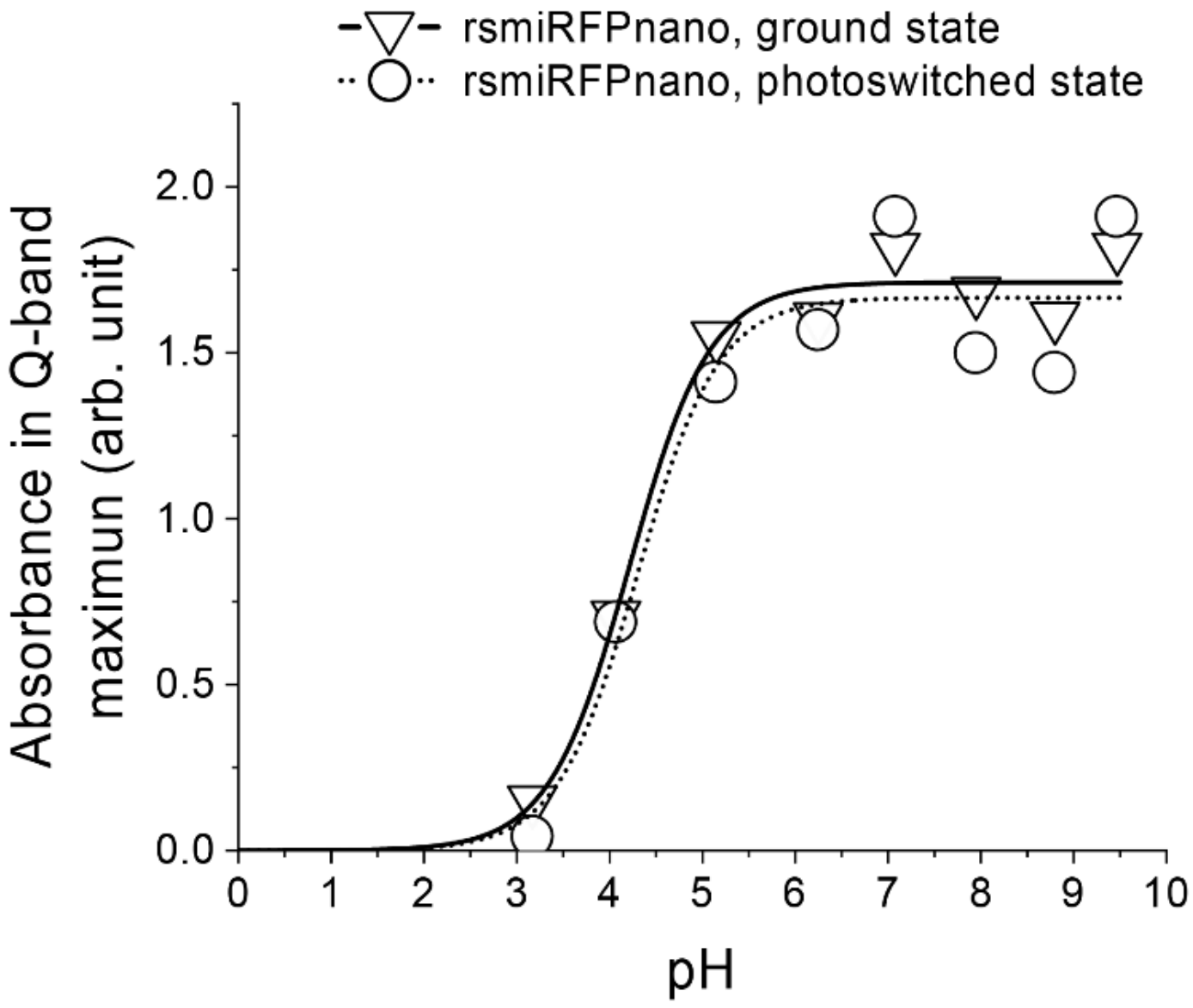

Supplementary Figure 4. Determination of p*K*a values for the ground and photoswitched states of rsmiRFPnano. Absorbance at the Q-band maximum is plotted as a function of pH for the ground state (716 nm, triangles, solid line) and the photoswitched state (650 nm, circles, dashed line). Individual data points were fitted using a sigmoidal function based on the Henderson–Hasselbalch equation, implemented in Origin software, yielding p*K*a values of 4.2 and 4.3 for the ground and photoswitched states, respectively.

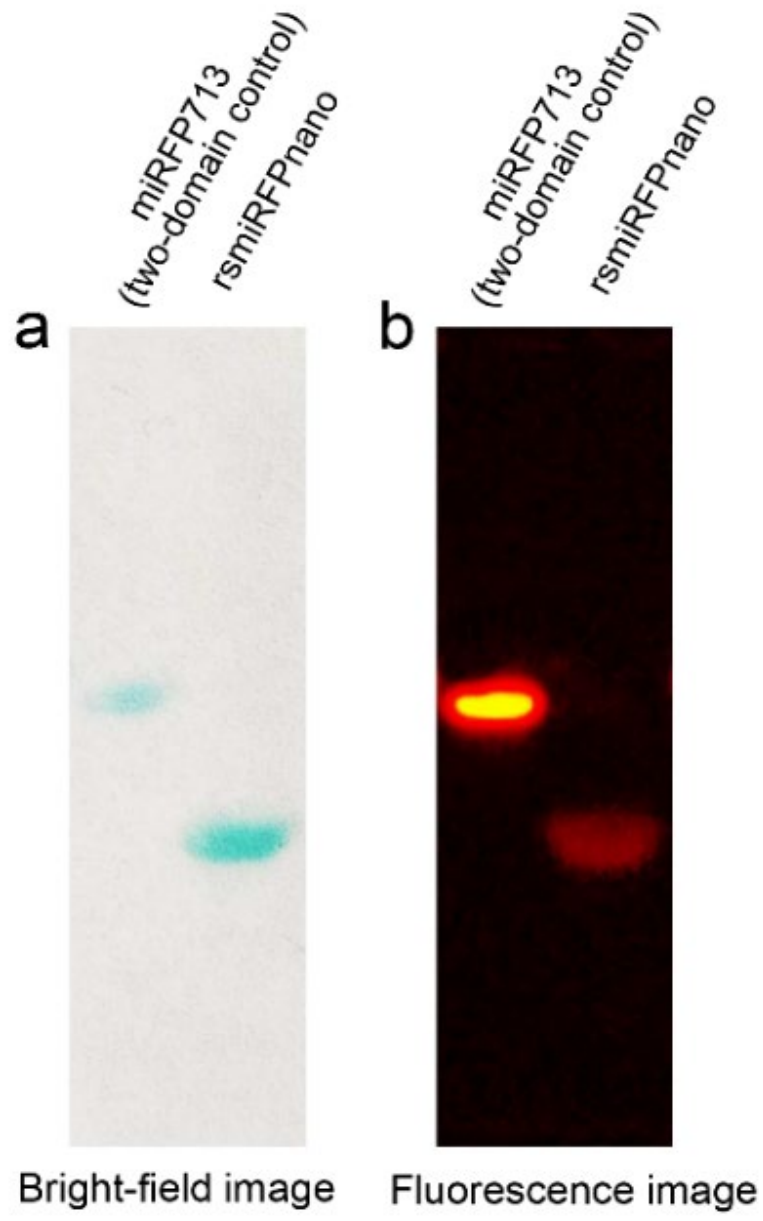

Supplementary Figure 5. Unstained native PAGE gel. (a) Daylight image of a polyacrylamide gel loaded with 37 µg of undenatured miRFP713 consisting of the PAS-GAF domains (left lane; MW 34.6 kDa) and rsmiRFPnano, consisting of a single GAF domain (right lane; MW 18.8 kDa). **(b)** Fluorescence imaging of the same gel shown in (a) was acquired using a Newton FT-900 imaging system with excitation at 680/30 nm and a 750 nm long-pass emission filter.

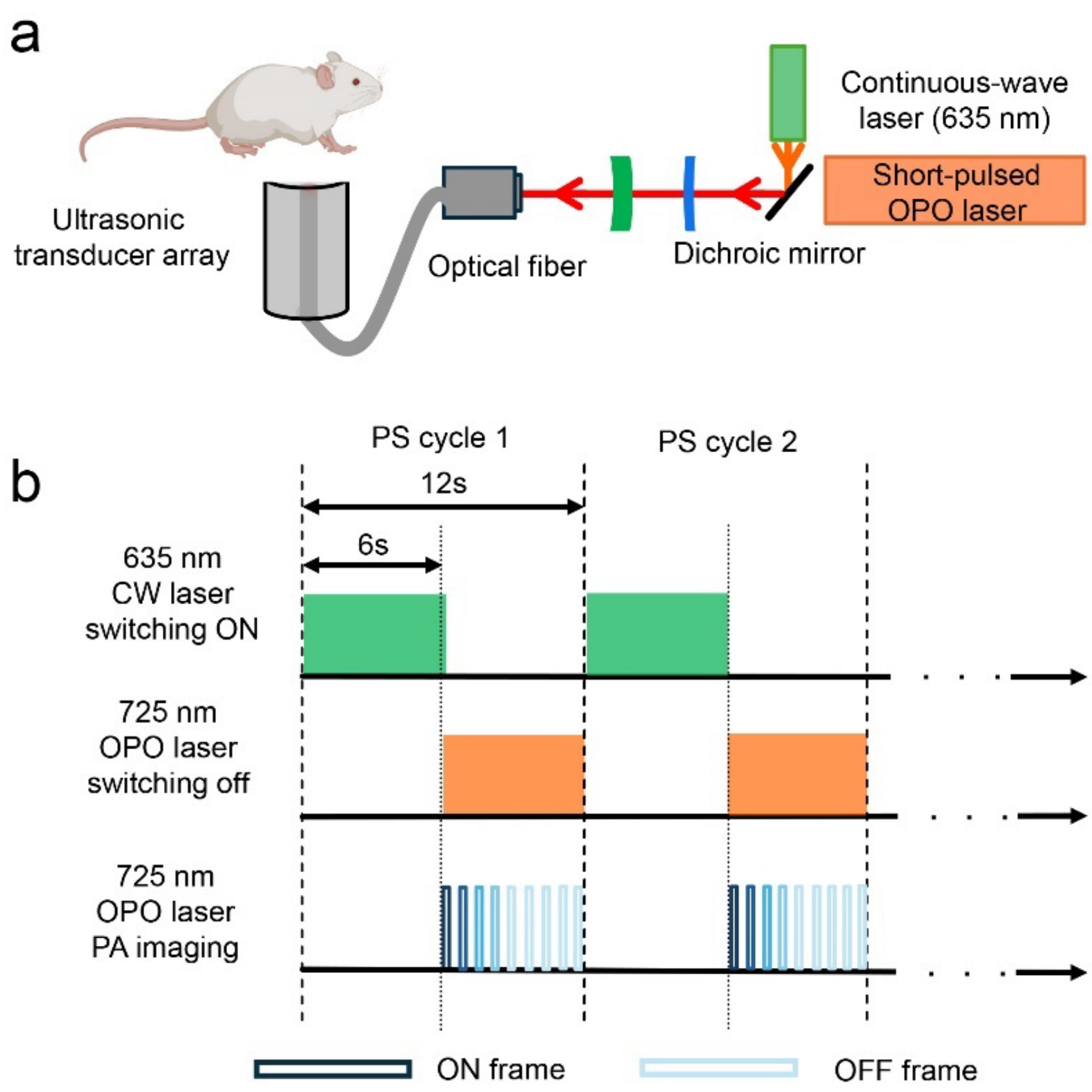

Supplementary Fig. 6. Schematic of the differential PAT system. (a) Schematic of the system setup. A short-pulsed optical parametric oscillator (OPO) laser for photoacoustic excitation and a continuous-wave (CW) laser for photoswitching are combined using a dichroic mirror and delivered to the animal through an optical fiber bundle. Photoacoustic signals are detected by a hemi-spherical ultrasonic transducer array. **(b)** Sequence of the photoswitching and PA imaging of the rsmiRFPnano. Each photoswitching (PS) cycle consists of alternating the laser illumination at 635 nm and pulsed OPO excitation at 725 nm. Photoacoustic images acquired during the ON and OFF states are alternately recorded and subsequently used to calculate differential photoacoustic signals by subtracting the OFF-state images from the ON-state images.

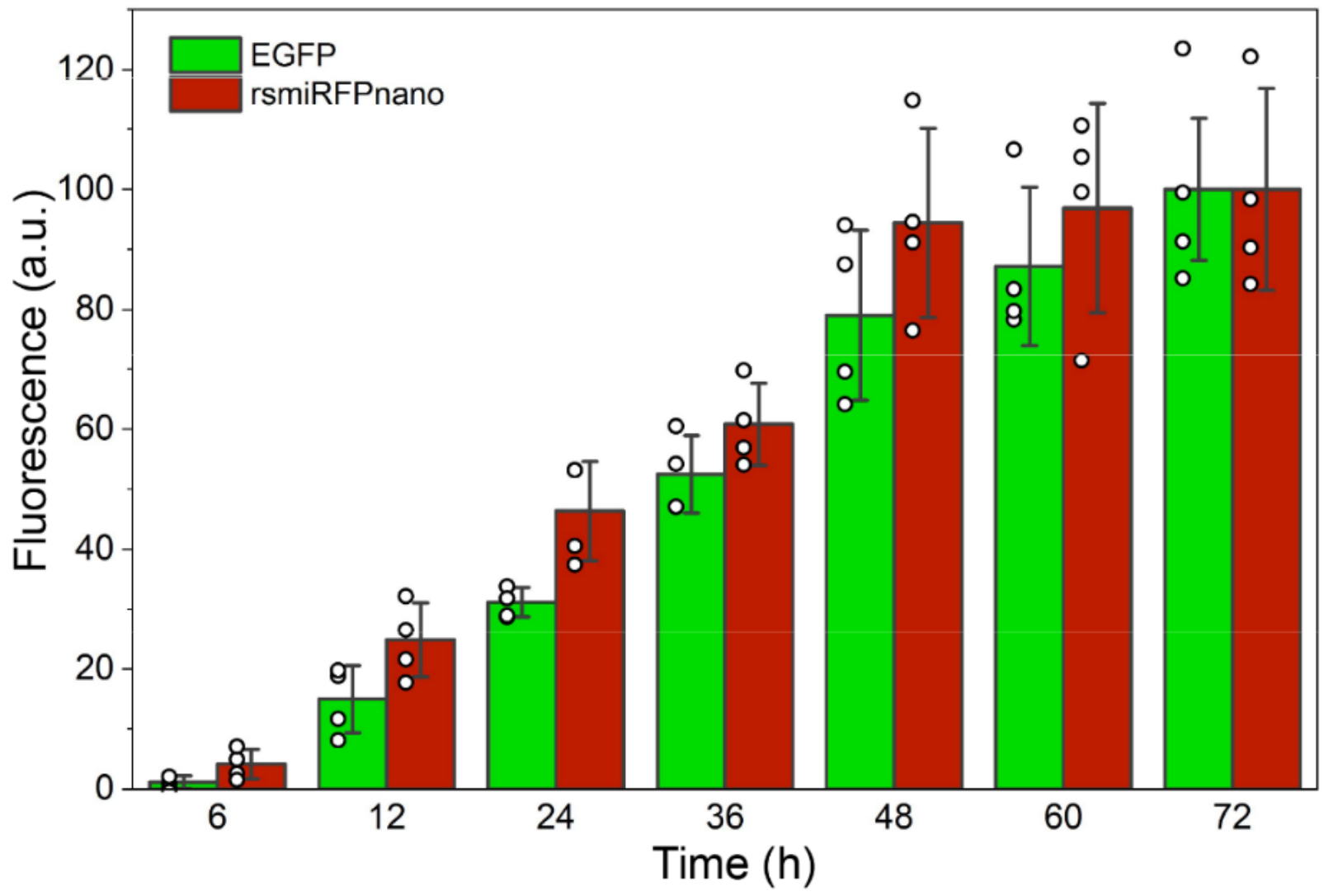

Supplementary Fig. 7. Time course of doxycycline-induced rsmiRFPnano and EGFP expression. HEK293T cells were co-transfected with pTRE-EGFP, pTRE-rsmiRFPnano, and Tet-On plasmids at a ratio of 1:6:3. Cells were treated with 1 μg/mL doxycycline at time "0" and analyzed at the indicated time points. Fluorescence was measured by flow cytometry (green columns – EGFP; dark-red columns – rsmiRFPnano). For each protein, fluorescence values of untreated cells were subtracted, and the highest value was set to 100%. Data are presented as mean ± s.d.; n = 4 independent experiments.

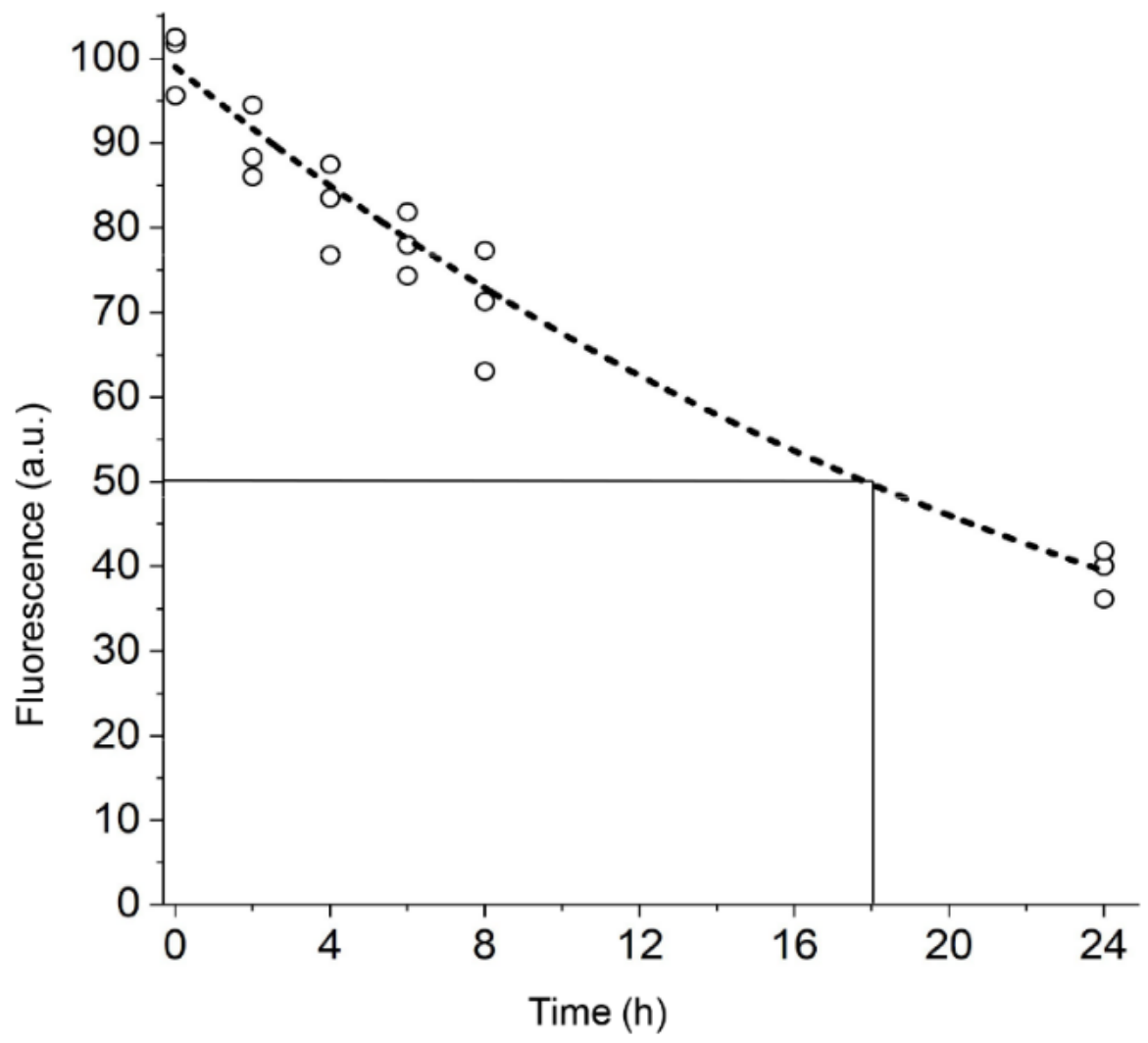

Supplementary Fig. 8. rsmiRFPnano stability in HEK-293T cells. Cells expressing rsmiRFPnano were treated with 50 μg ml^{-1} cycloheximide to inhibit protein synthesis, and fluorescence was measured by flow cytometry at the indicated time points. Background-subtracted fluorescence values were normalized to the 0 h time point and fitted to a single exponential decay model with the baseline constrained to zero, yielding an apparent half-life of 18 h.

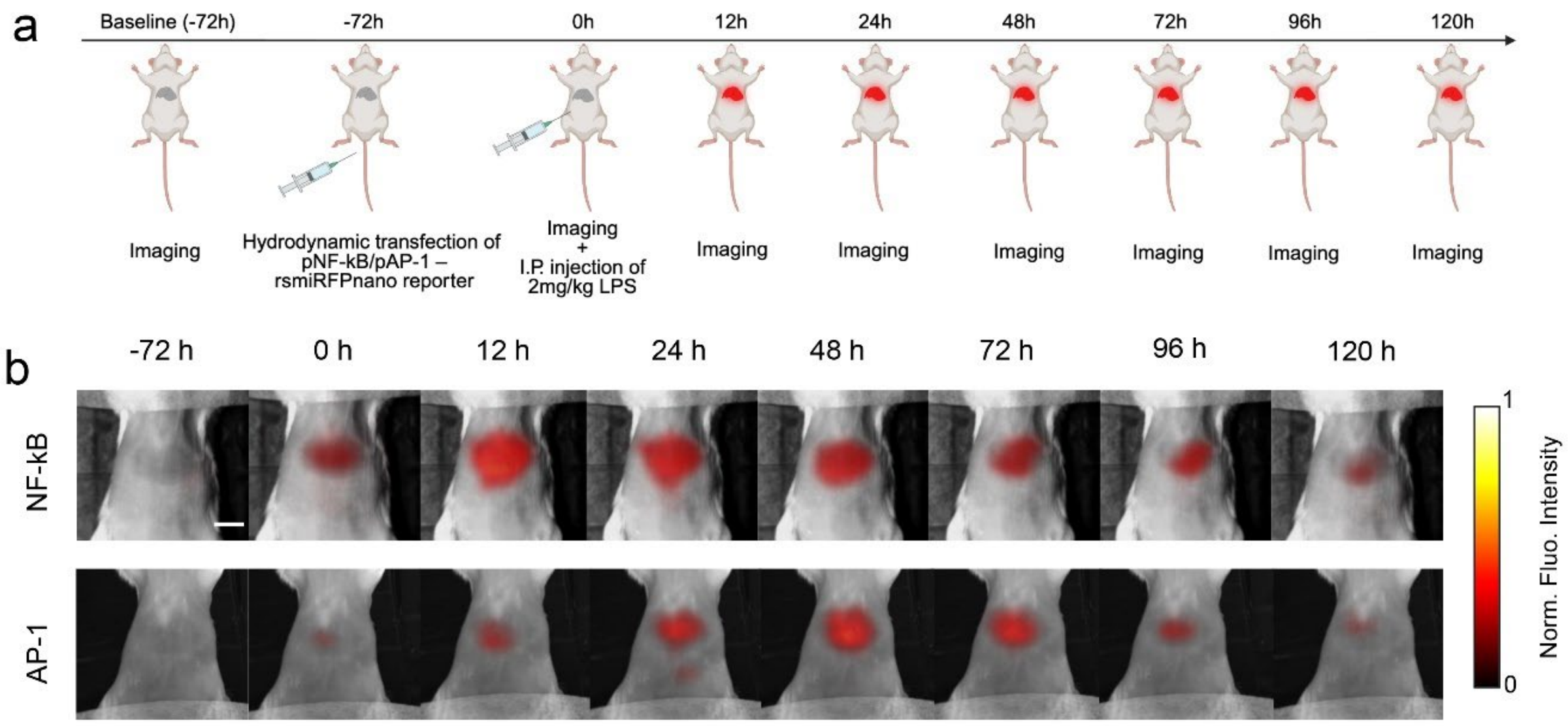

Supplementary Fig. 9. LPS-induced liver inflammation mouse model. (a) Schematic of the LPS-induced inflammation mouse model and imaging timeline. Baseline imaging was performed 72 h prior to the LPS treatment, followed by serial imaging from 0 to 120 h post LPS treatment. Created in BioRender. Xu, Y. (2026) https://BioRender.com/id584r1. **(b)** Representative fluorescence images of mice expressing NF-κB or AP-1 reporters in the livers at the indicated time points. Images are representative of *n* = 3 mice. Liver-confined fluorescence signals increased following LPS challenge and exhibited distinct temporal activation profiles. Fluorescence intensity was normalized to the all-time maximum for better visualization. The excitation light source was a 680 nm laser diode (M660L4, Thorlabs) with a 680/10 nm bandpass filter (FB680/10, Thorlabs). The surface excitation light intensity was ~50 mW cm^{-2}, and the camera exposure time was 20 ms. Fluorescence emission was collected using a 750/40 nm bandpass filter (FB750-40, Thorlabs). Scale bar, 10 mm.

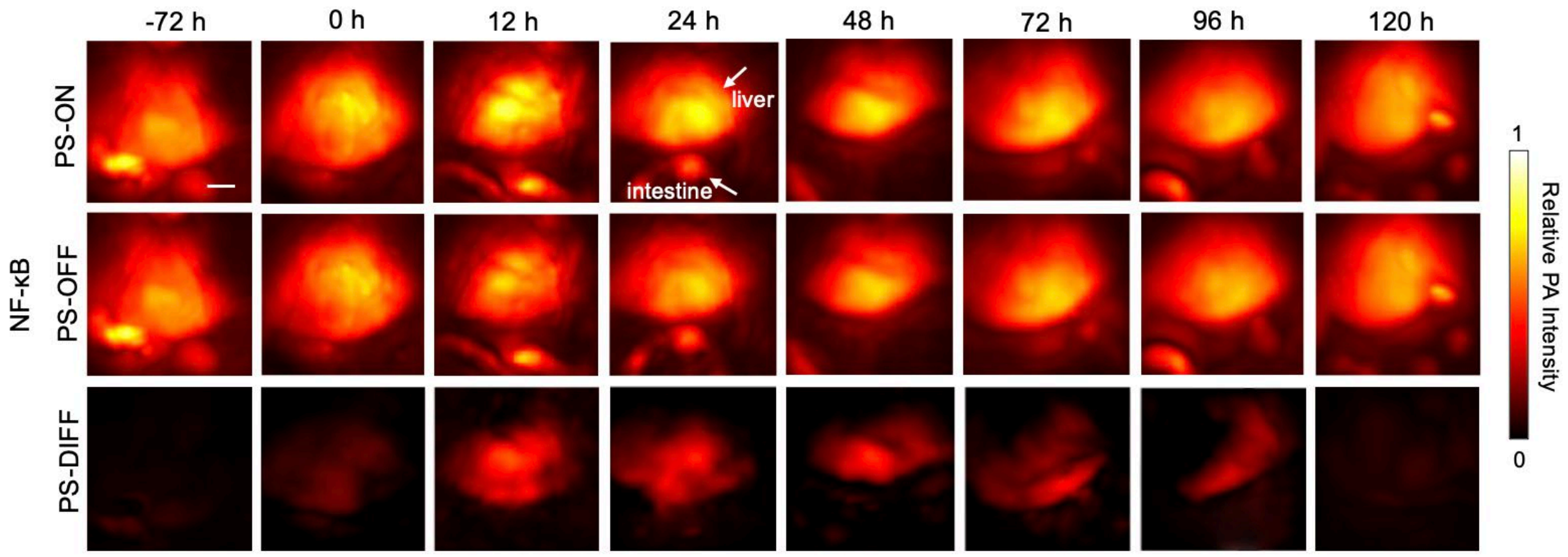

Supplementary Fig. 10. Photoacoustic imaging of rsmiRFPnano-based NF-kB reporters in mice upon LPS treatment. Representative ON-state (PS-ON), OFF-state (PS-OFF), and differential PAT (PS-DIFF) images showing NF-κB reporter activation in the liver at different time points before and after LPS treatment. Images are representative of $n = 3$ mice. Both ON-state and OFF-state PAT images contain PA signals from both liver and intestinal regions (arrows). In contrast, differential PAT selectively preserves the photoswitchable rsmiRFPnano signals expressed in the liver while substantially suppressing non-switching background. All images were displayed using the same relative PA intensity scale. Scale bars, 2 mm.

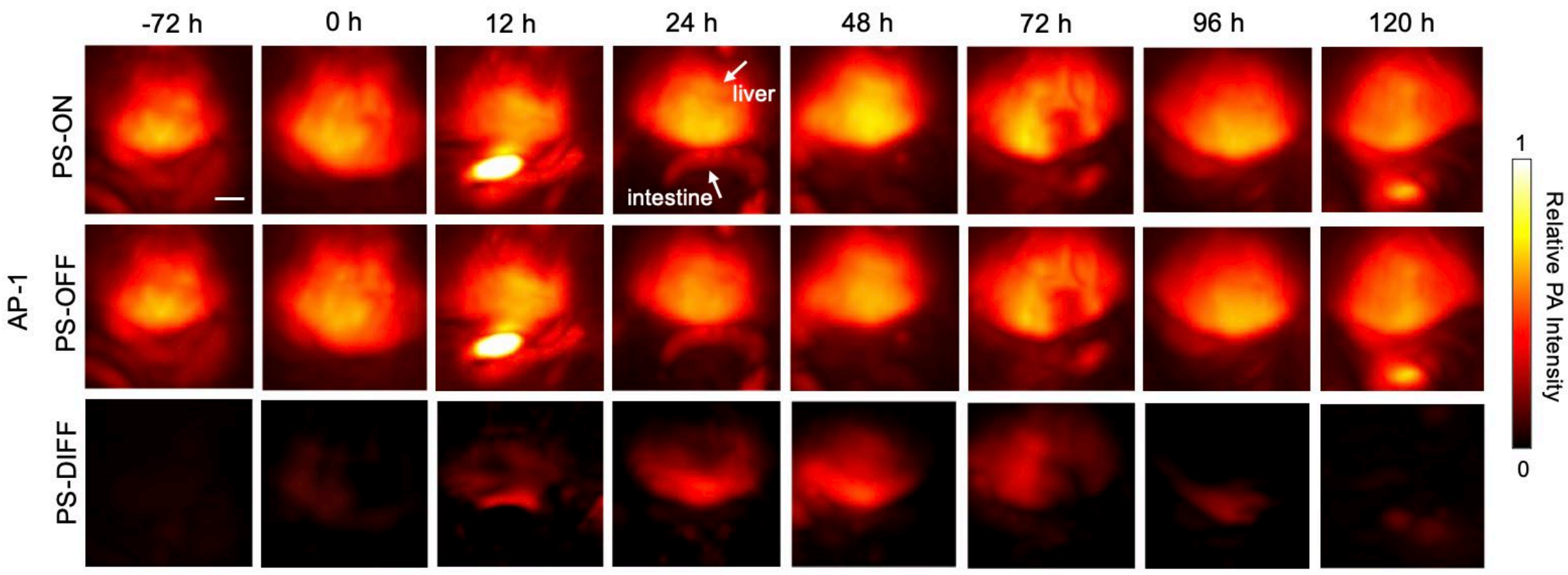

Supplementary Fig. 11. Photoacoustic imaging of rsmiRFPnano-based AP-1 reporters in mice upon LPS treatment. Representative ON-state (PS-ON), OFF-state (PS-OFF), and differential PAT (PS-DIFF) images showing AP-1 reporter activation in the liver at different time points before and after LPS treatment. Images are representative of n = 3 mice. Both ON-state and OFF-state PAT images contain PA signals from both liver and intestinal regions (arrows). In contrast, differential PAT selectively preserves the photoswitchable rsmiRFPnano signals expressed in the liver while substantially suppressing non-switching background. All images were displayed using the same relative PA intensity scale. Scale bars, 2 mm.

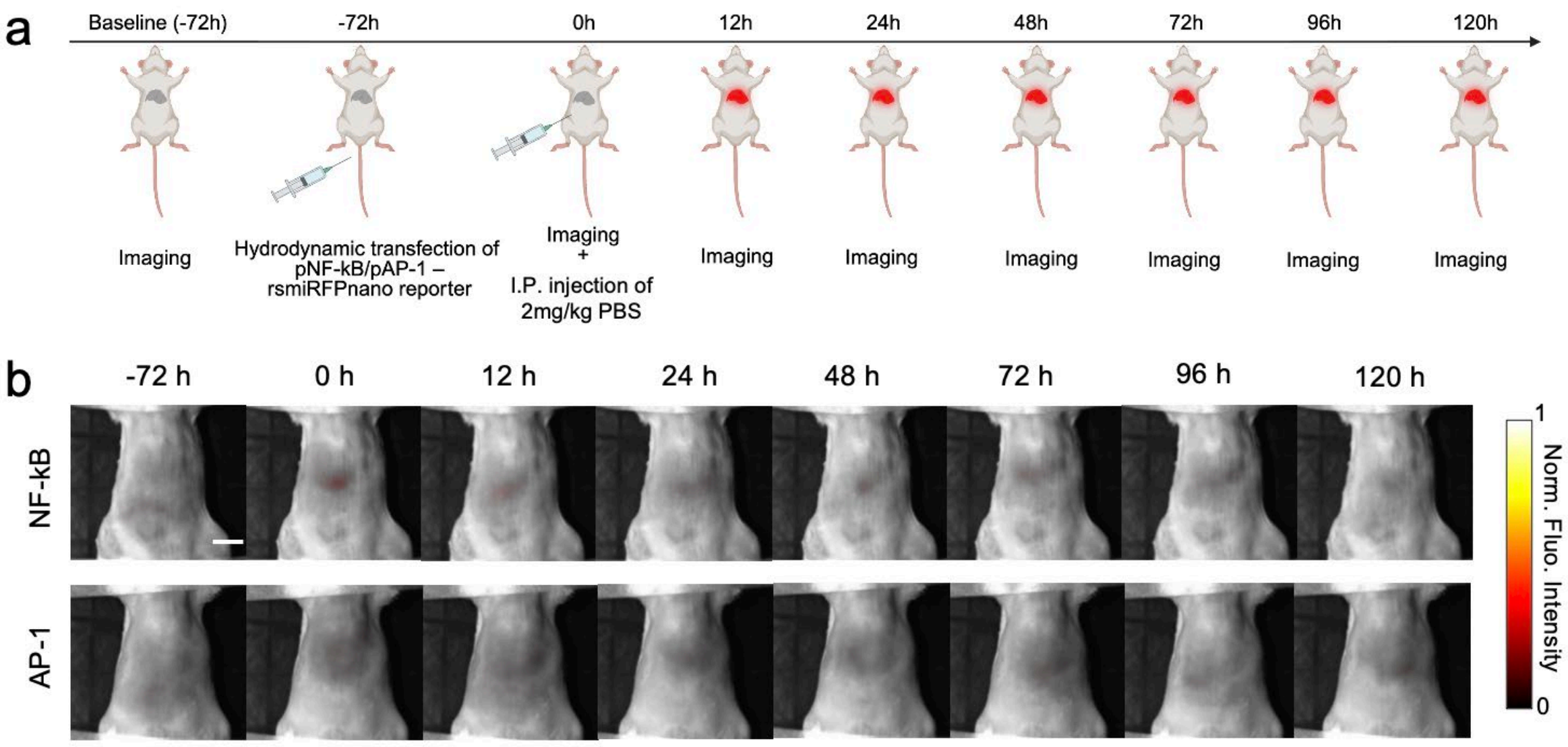

Supplementary Fig. 12. PBS-treated control mouse model. (a) Schematic of the PBS-treated control experiment and imaging timeline. Baseline imaging was performed 72 h prior to PBS injection, followed by serial imaging from 0 to 120 h post treatment. Mice received the same hydrodynamic transfection of NF-κB or AP-1 rsmiRFPnano reporter constructs as the LPS-treated groups. Created in BioRender. Xu, Y. (2026) https://BioRender.com/id584r1. **(b)** Representative fluorescence images of mice expressing NF-κB or AP-1 reporters at the indicated time points following PBS injection. Images are representative of *n* = 3 mice. Liver-localized fluorescence signals were weak following PBS treatment, in contrast to the sustained activation observed following LPS challenge (Supplementary Fig. 9). Fluorescence intensity was normalized for visualization. Scale bar, 10 mm.

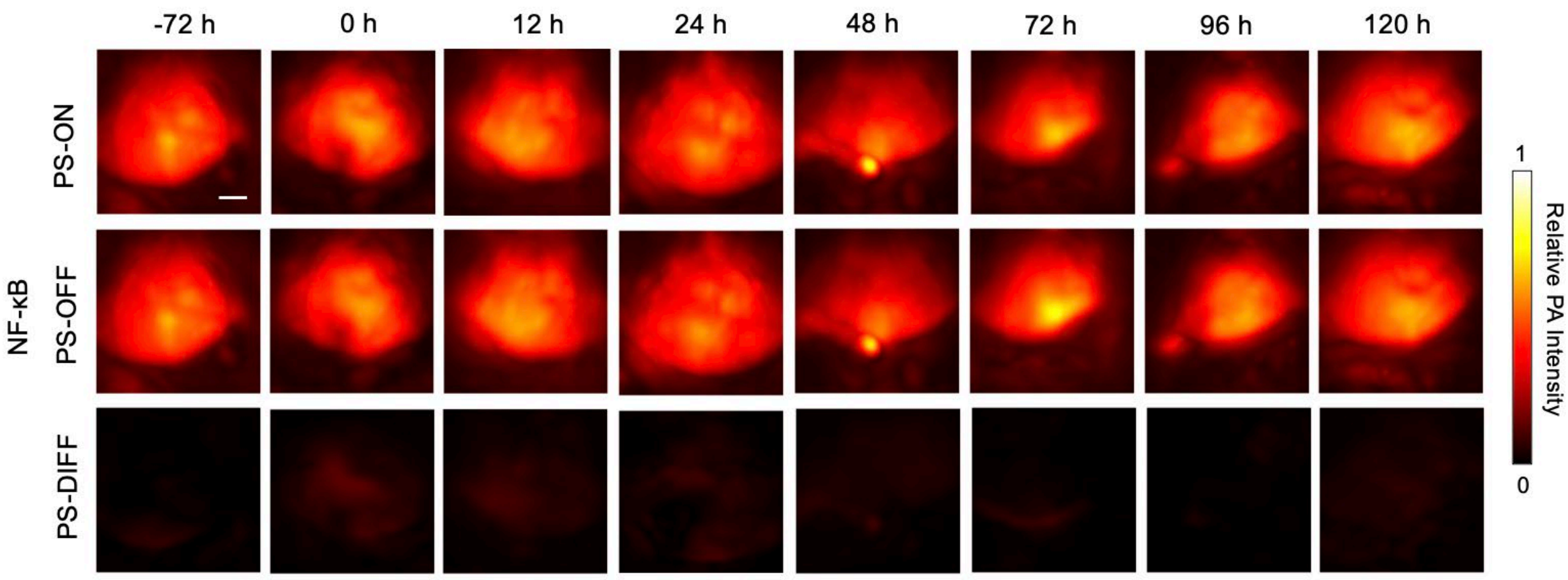

Supplementary Fig. 13. Photoacoustic imaging of rsmiRFPnano-based NF-κB reporters in mice following PBS treatment. Representative ON-state (PS-ON), OFF-state (PS-OFF), and differential PAT (PS-DIFF) images showing NF-κB reporter activity in the liver at different time points before and after PBS treatment. Images are representative of $n = 3$ mice. In contrast to the LPS-treated animals (Supplementary Fig. 10), differential PAT images showed only weak transient signals that progressively decreased over time and did not exhibit sustained reporter activation. All images were displayed using the same relative PA intensity scale. Scale bar, 2 mm.

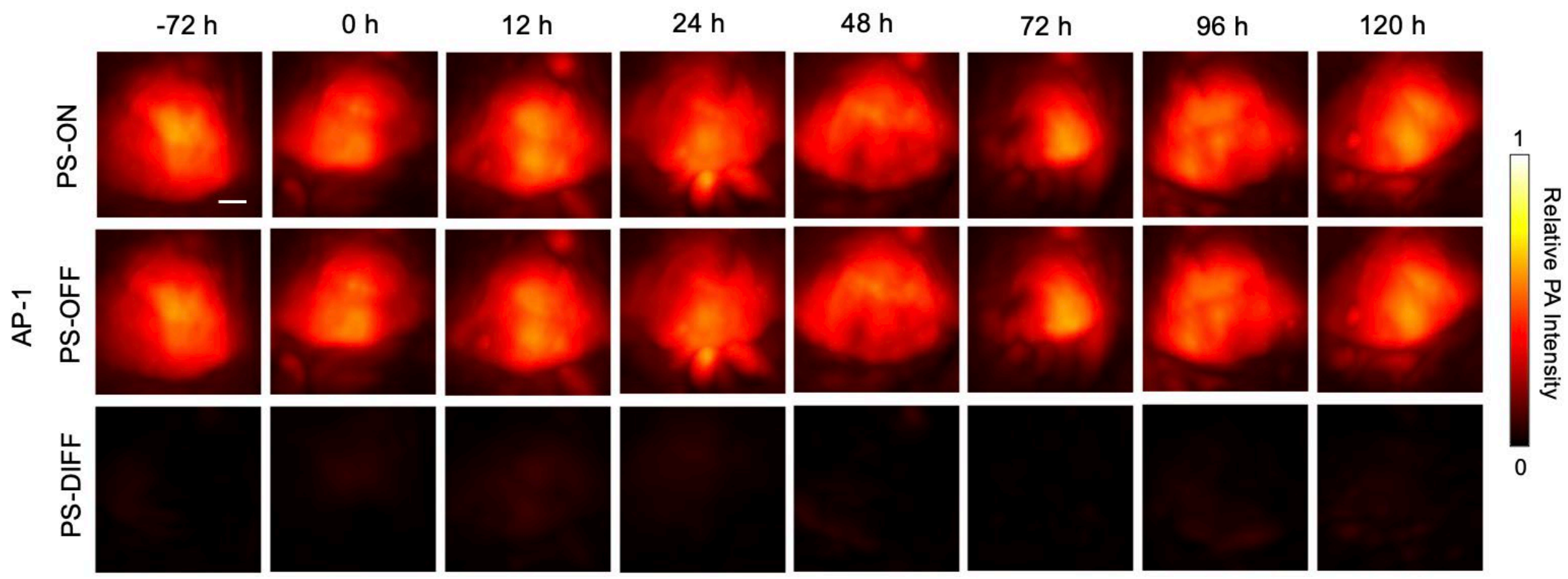

Supplementary Fig. 14. Photoacoustic imaging of rsmiRFPnano-based AP-1 reporters in mice following PBS treatment. Representative ON-state (PS-ON), OFF-state (PS-OFF), and differential PAT (PS-DIFF) images showing AP-1 reporter activity in the liver at different time points before and after PBS treatment. Images are representative of n = 3 mice. In contrast to the LPS-treated animals (Supplementary Fig. 11), differential PAT images remained near background levels throughout the imaging period. All images were displayed using the same relative PA intensity scale. Scale bar, 2 mm.

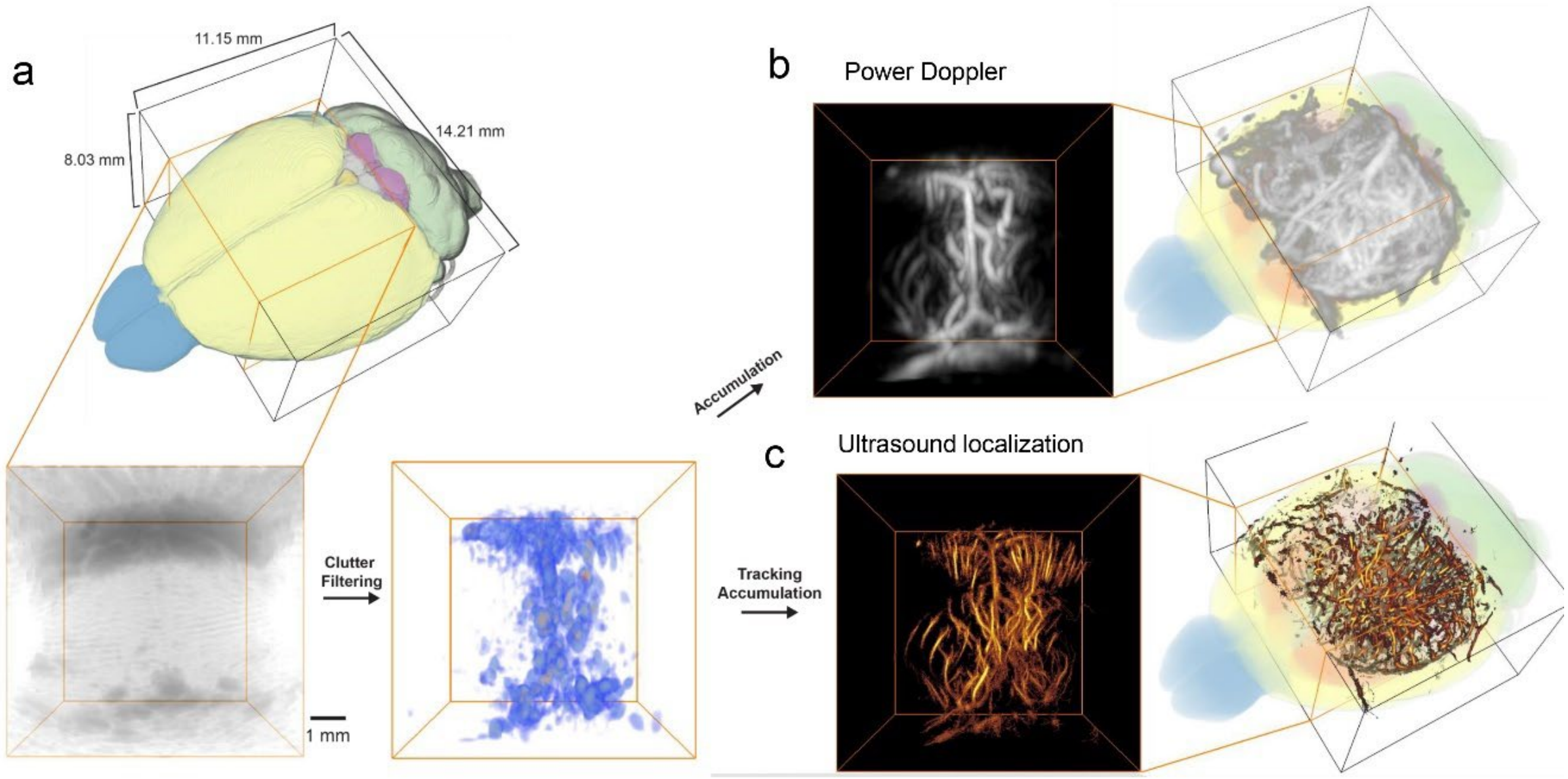

Supplementary Fig. 15. 3D-ULM processing workflow. (a) Ultrasound (US) B-mode images are reconstructed by using the 3D delay and sum method (DAS), and spatial-temporal singular value decomposition (SVD) filtering is applied to remove the clutter signals. **(b)** Power Doppler image is formed by accumulating the SVD filtered images. **(c)** Radial symmetry-based localization is performed to extract the central positions of microbubble signals. Trajectories retrieved using the 3D tracking algorithm are accumulated to form the ULM density map and flow speed map.

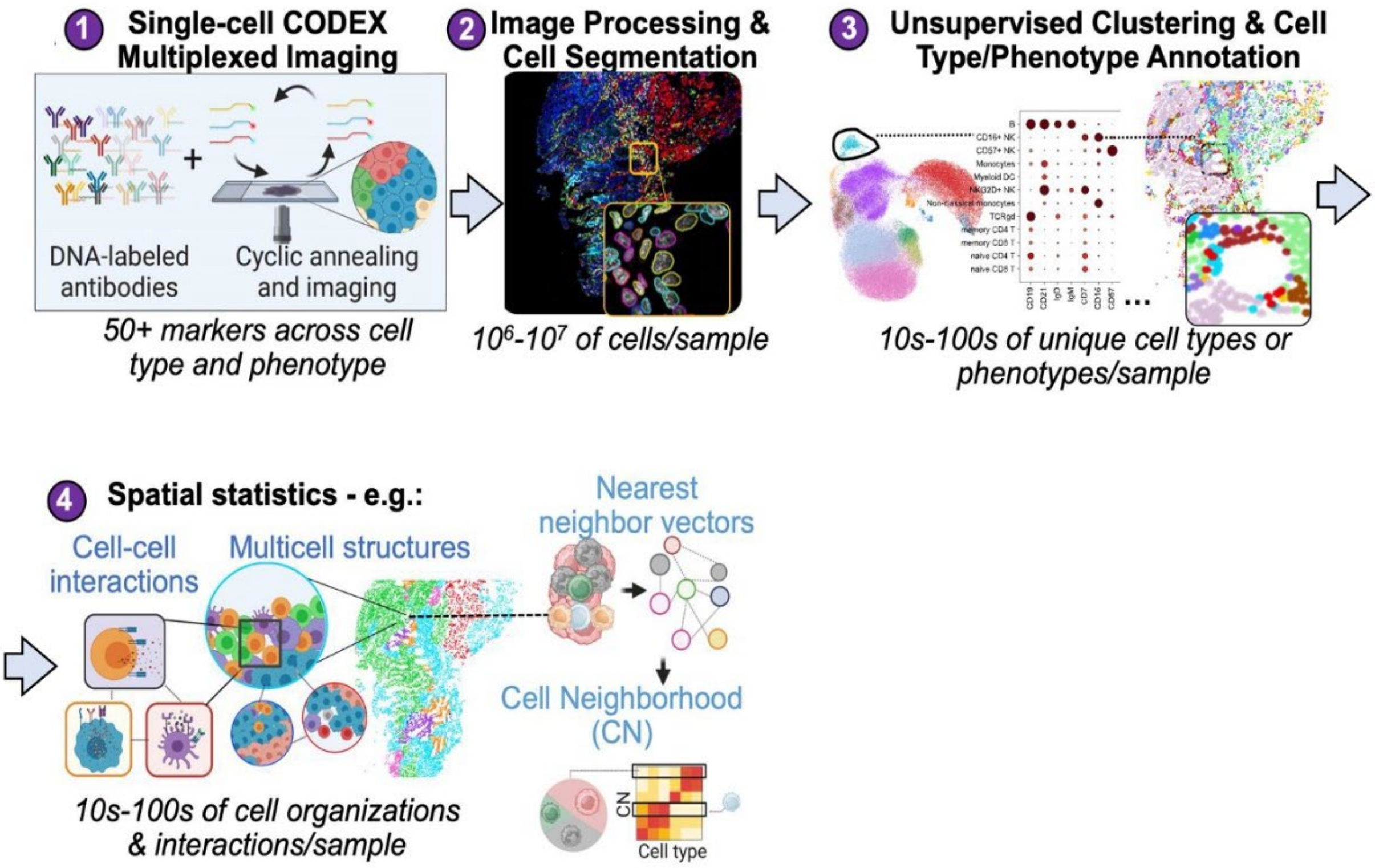

Supplementary Fig. 16. Schematic overview of the CODEX multiplexed imaging and analysis workflow. Step 1: DNA-barcoded antibodies are cyclically annealed and imaged to enable simultaneous detection of >50 protein markers at single-cell resolution. **Step 2:** Images are processed for cell segmentation. **Step 3:** Unsupervised clustering and cell type or phenotype annotation. **Step 4:** Spatial statistics, including cell–cell interactions, multicellular structures, and cell neighborhood (CN) analysis, are subsequently performed.

57 marker CODEX panel

			Neutrophil	*T cell*
	Inflammation	*B cell*	Ly6G	CD3
Tumor	PDL1	B220	CD11b	CD8
CD71	IL10	CD138	CD45	CD4
Ki67	Granzyme B	CD38		FoxP3
PanCK	CXCL10	CD19	*NK*	CD27
	PD1	CD79b	NKp46	CD25
Stromal	Tim3	IgD	KLRG1	TCRb
aSMA	ICOS	CD45	NK1.1	CD69
FAP	IFNg	Lysozy	NKG2A	CCR7
CD44	MHC-I			CD62L
CD90		*Macrophage*	*Vessel*	TCF1/7
Vimentin	*DC*	16/32	CD31	CD103
Ly6C	CD11c	F4/80	CD34	
keratin8	MHCII	CD169	CD36	
CD117	IRF8	CD163	CD106	
Sca-1	CD86	CD206	PDPN	

Supplementary Fig. 17. Composition of the 57-marker CODEX antibody panel used for cell type and functional state profiling. The panel includes markers for epithelial, stromal, vascular, and immune cell populations, as well as functional markers associated with proliferation and immune activation.

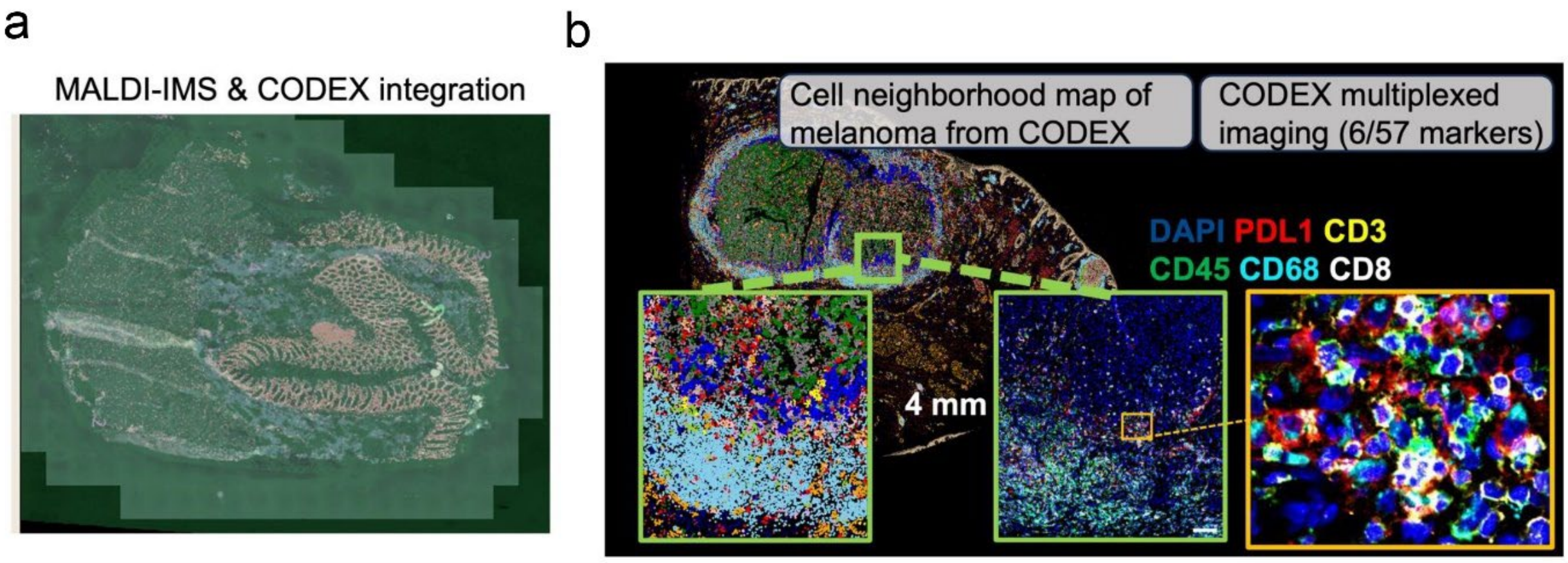

Supplementary Fig. 18. Spatial co-registration of MALDI-IMS and CODEX imaging with cell neighborhood analysis. (a) Representative example of spatial integration between MALDI imaging mass spectrometry (MALDI-IMS) and CODEX multiplexed imaging, demonstrating co-registration of molecular and protein-level spatial information within the same tissue section. **(b)** Example of cell neighborhood analysis derived from CODEX imaging of a melanoma tissue section. Left, whole-section cell neighborhood map generated from single-cell spatial data. Right, corresponding CODEX multiplexed immunofluorescence images showing selected markers at increasing magnification. Scale bars, 4 mm (whole section).

rsNIR-Fb$_{GFP}$:

MDQVQLVESGGALVQPGGSLRLSCAASGFPVNRYSMRWYRQAPGKEREWVAGMSSAGDRSSYEDSGGGGSMADLESK
FSTLTQEIRRLLDIERVTIYKFREDYFGDFITESEAGGWRKLVGSGWEDPYLNEHQGGRFQQNQPFVVDDIYLGETIWEEGKF
NLQKPKRPLTDCHIEALESFEVKSCAVVAIFQGQKLWGLLSAFQNSAPRHWDEAEVQLLMRTADQLGVIIQQAGGGGSKGR
FTISRDDARNTVYLQMNSLKPEDTAVYYCNVNVGFEYWGQGTQVTVSS

rsNIR-Fb$_{gp41}$:

MDVQLQESGGGLVQPGGSLRLSCAASGNIVSIDAAGWFRQAPGKQREPVATILTGGATNYADSGGGGSMADLESKFSTLT
QEIRRLLDIERVTIYKFREDYFGDFITESEAGGWRKLVGSGWEDPYLNEHQGGRFQQNQPFVVDDIYLGETIWEEGKFNLQK
PKRPLTDCHIEALESFEVKSCAVVAIFQGQKLWGLLSAFQNSAPRHWDEAEVQLLMRTADQLGVIIQQAGGGGSVKGRFTIS
RDNAKNTVYLQMNSLKPEDTAVYYCYAPMIYYGGRYSDYWGQGTQVTVSS

rsNIR-Fb$_{RBD}$:

MQVQLVESGGGLVQAGGSLRLSCAVSGLGAHRVGWFRRAPGKEREFVAAIGANGGNTNYLDSGGGGSMADLESKFSTLT
QEIRRLLDIERVTIYKFREDYFGDFITESEAGGWRKLVGSGWEDPYLNEHQGGRFQQNQPFVVDDIYLGETIWEEGKFNLQK
PKRPLTDCHIEALESFEVKSCAVVAIFQGQKLWGLLSAFQNSAPRHWDEAEVQLLMRTADQLGVIIQQAGGGGSVKGRFTIS
RDNAKNTIYLQMNSLKPQDTAVYYCAARDIETAEYTYWGQGTQVTVSS

Supplementary Fig. 19. Amino acid sequences of rsNIR-Fbs specific for EGFP, HIV-1 gp41 protein, and the SARS-CoV-2 spike RBD domain. Nanobody sequences are shown in blue, linker sequences in grey, and the inserted rsmiRFPnano sequence in red.

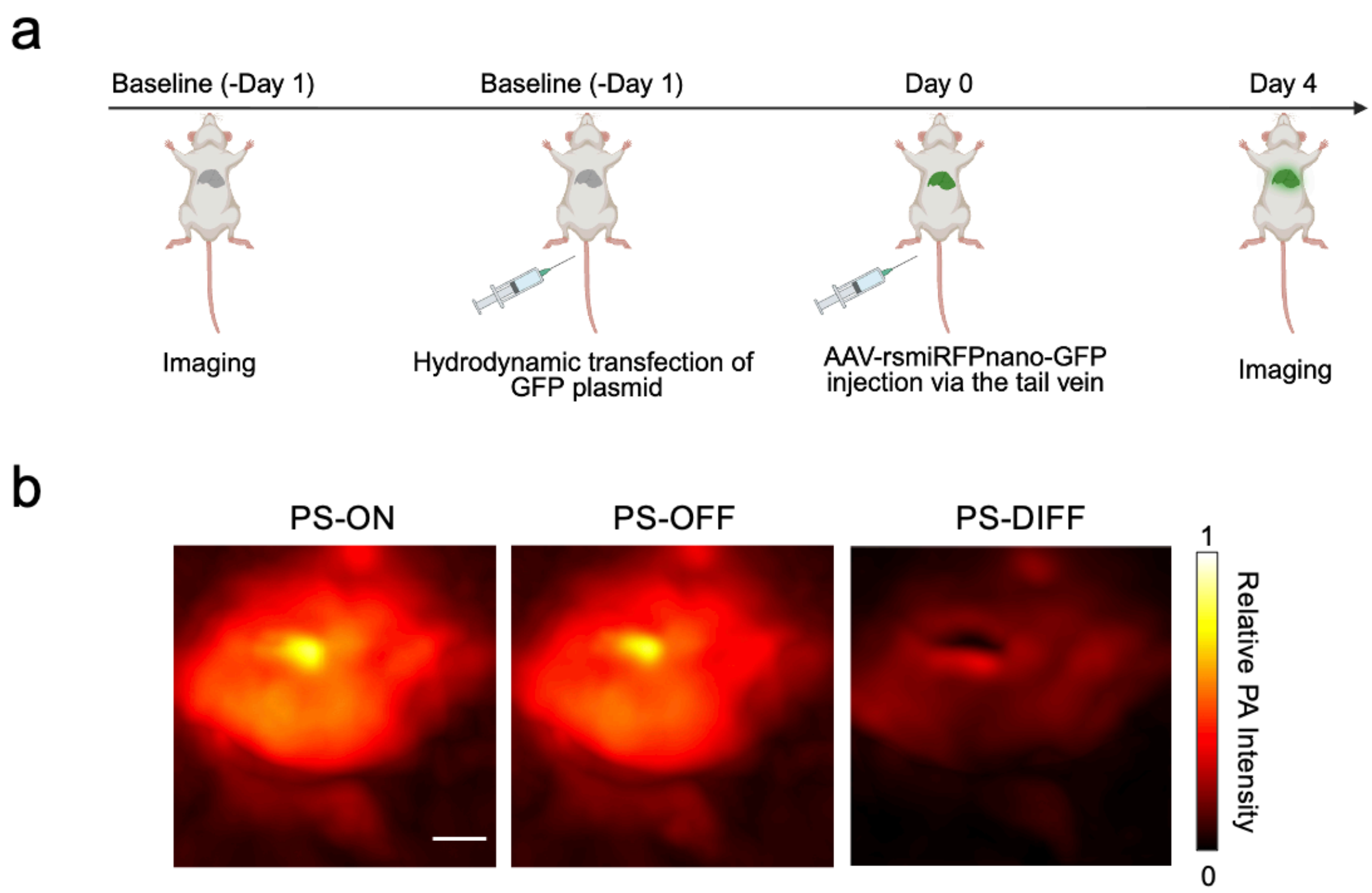

Supplementary Fig. 20. Schematic of the *in vivo* experimental workflow for antigen-dependent rsNIR-Fb activation in the mouse liver. **(a)** An EGFP-encoding pcDNA3.1-EGFP plasmid was first hydrodynamically transfected to induce a liver-confined EGFP expression. 24 hours later, AAV particles encoding rsNIR-Fb$_{GFP}$ under the CAG promoter with wide tissue expression were systemically administered. *In vivo* differential molecular PAT imaging was subsequently performed 96 hours after the AAV delivery. Created in BioRender. Xu, Y. (2026) https://BioRender.com/sm0548u. **(b)** Representative ON-state (PS-ON), OFF-state (PS-OFF), and differential PAT (PS-DIFF) images of the mouse liver at day 4 after AAV injection. Images are representative of n = 3 mice. All images were displayed using the same relative PA intensity scale. Scale bar, 2 mm.

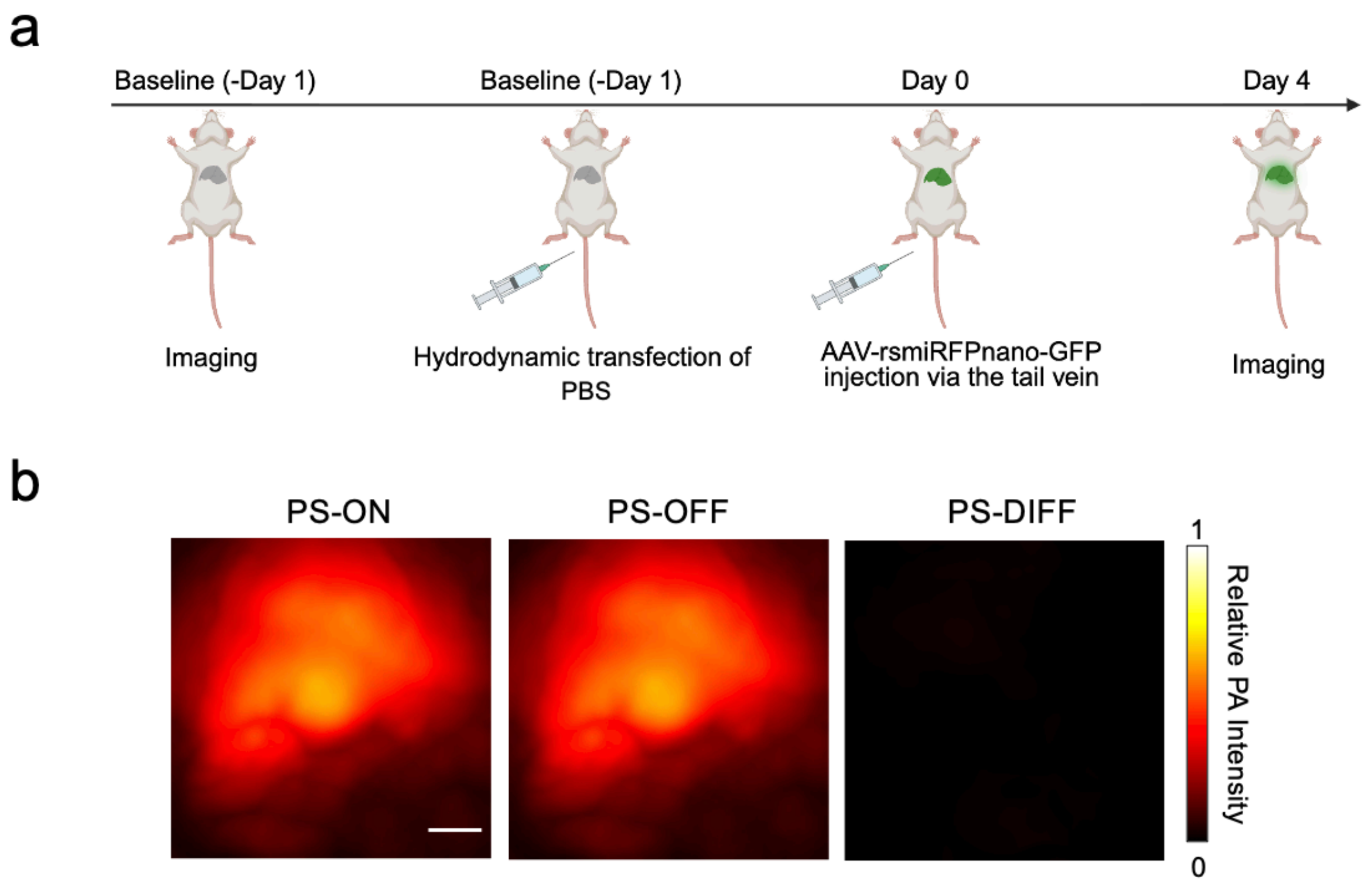

Supplementary Fig. 21. Schematic of the negative-control workflow for in vivo rsNIR-Fb imaging in the mouse liver. (a) Mice received PBS instead of the EGFP-encoding pcDNA3.1-EGFP plasmid during the hydrodynamic injection step. 24 hours later, AAV particles encoding rsNIR-FbGFP under the CAG promoter with wide tissue expression were systemically administered. In vivo differential molecular PAT imaging was subsequently performed 96 hours after the AAV delivery. Created in BioRender. Xu, Y. (2026) https://BioRender.com/sm0548u. **(b)** Representative ON-state (PS-ON), OFF-state (PS-OFF), and differential PAT (PS-DIFF) images of the mouse liver at day 4 after AAV injection. . Images are representative of $n = 3$ mice. All images were displayed using the same relative PA intensity scale. Scale bar, 2 mm.

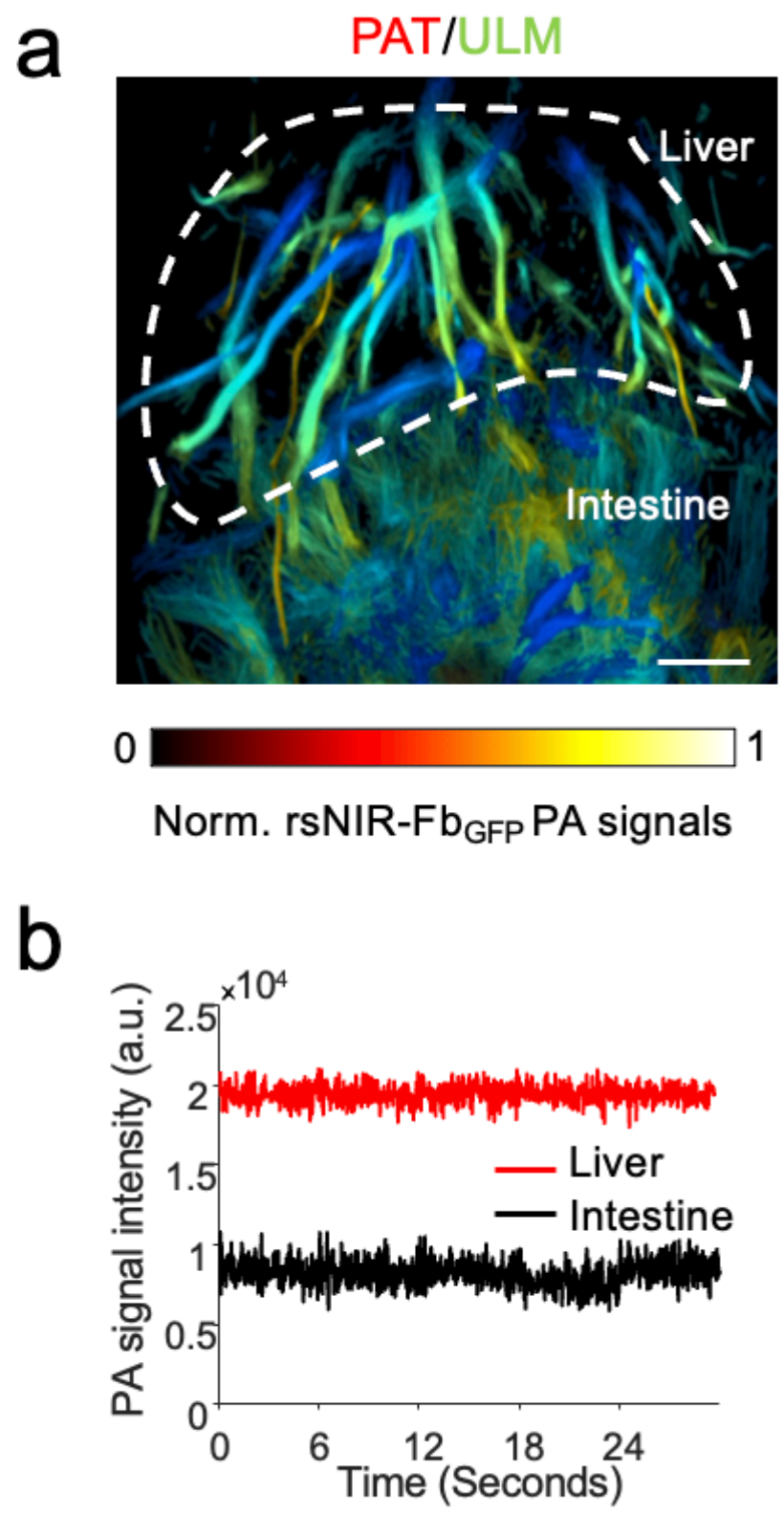

Supplementary Fig. 22. Negative control for antigen-dependent rsNIR-FbGFP activation in vivo. (a) Representative in vivo hybrid differential PAT/ULM image acquired from a mouse receiving systemic delivery of rsNIR-FbGFP in the absence of EGFP expression. Images are representative of n = 3 mice. No detectable differential PAT signals were observed in either the liver or intestine regions. Scale bar, 2 mm. **(b)** Dynamic photoacoustic signals extracted from the liver and intestine regions shown in (a). Neither region exhibited detectable photoswitching-associated PA modulation, indicating the absence of rsNIR-FbGFP stabilization without the target antigen.

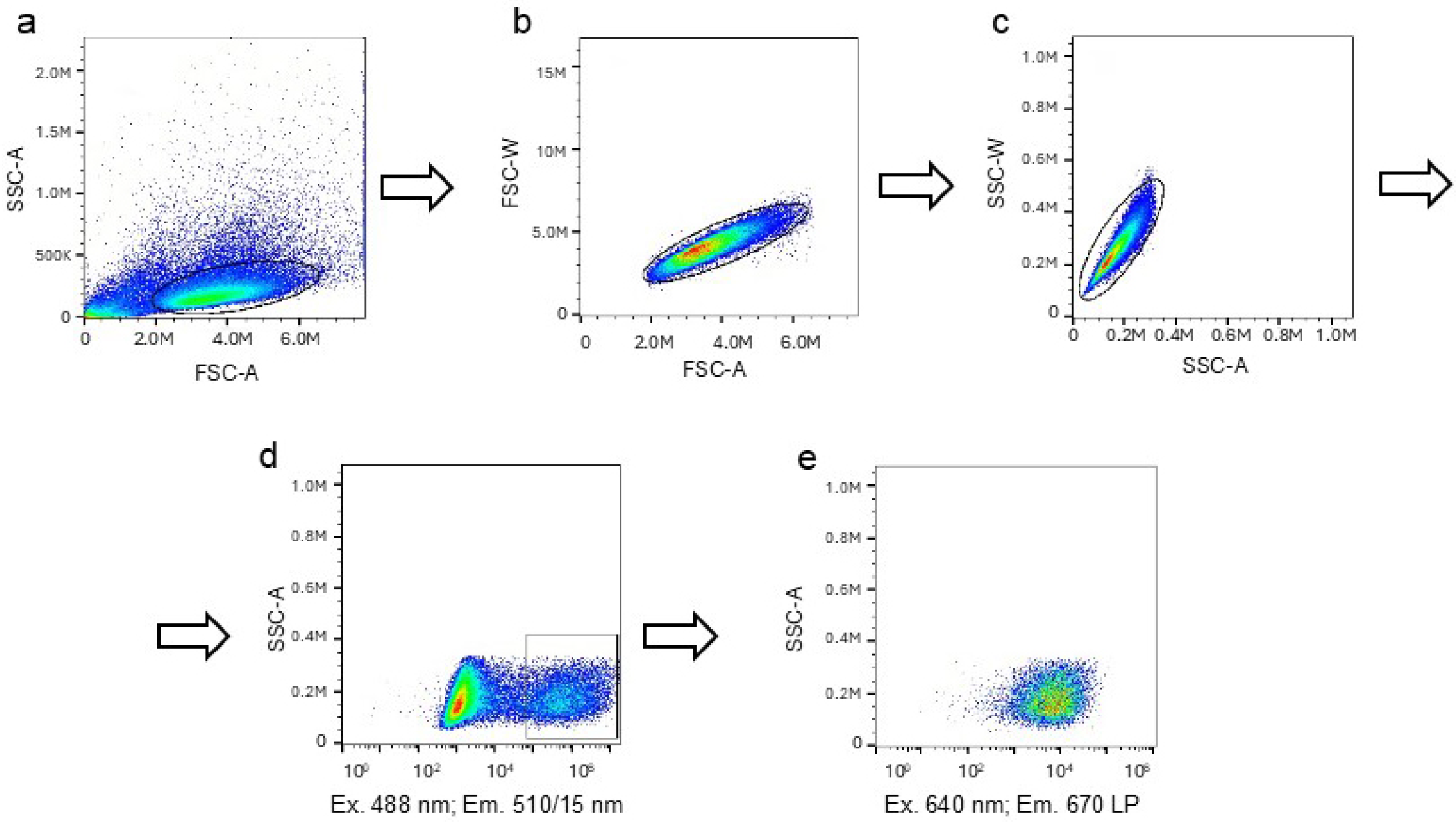

Supplementary Figure 23. Exemplifying gating strategy of flow cytometry analysis. Gating was performed using three gates, for (a) intact cells, (b) single cells, and (c) live cells. These cells were then gated against non-transfected cells in green (Ex. 488 nm, Em. 510/15 nm) channel for selecting EGFP positive cells (d). The latter cells were used to access the brightness in NIR (Ex. 640 nm, Em. 670 nm long-pass) channel (e).

Supplementary Table 1. Comparison of single-domain cyanobacteriochrome (CBCR)-derived photoswitchable photoacoustic protein rsmiRFPnano and constitutively fluorescent protein miRFP718nano.

Protein	GAF domain of parental CBCR	MW, kDa	Oligomeric state	Absorbance, nm	Extinction coefficient, $M^{-1}cm^{-1}$	p*K*a	Fluorescence excitation, nm	Fluorescence emission, nm	Fluorescence quantum yield, %	Reference
miRFP718nano	NpR3784	17.1	monomer	690	79,000	3.8	695	718	5.6	[1]
rsmiRFPnano	JSC1_58120_g3	18.8	monomer	ground state: 715	ground state: 85,000	ground state: 4.2	ground state: 715	ground state: 732	ground state: 0.25	this work
				photoswitched state: 650	photoswitched state: 88,000	photoswitched state: 4.3	photoswitched state: N.A.	photoswitched state: N.A.	photoswitched state: N.A.	

N.A. – not applicable

1. Oliinyk, O.S. *et al.* Deep-tissue SWIR imaging using rationally designed small red-shifted near-infrared fluorescent protein. *Nat Methods* **20**, 70-74 (2023).

Supplementary Table 2. Quantitative photoacoustic performance metrics of rsmiRFPnano in phosphate-buffered saline (PBS) and whole blood.

Parameters	rsmiRFPnano in blood	Blood only	rsmiRFPnano in PBS	PBS only
Mean Signal Intensity (a.u.). I_S	4008.58	290.91	2162.18	187.89
Mean Background Intensity (a.u.), I_B	134.14	66.78	63.89	63.47
Background Standard Deviation (a.u.), σ_B	74.03	63.48	33.26	37.11
Contrast Ratio, $(I_S - I_B) / I_B$	28.88	3.36	32.84	1.96
Signal-to-Noise Ratio (SNR), I_S / σ_B	54.15	4.58	65.01	5.06
Contrast-to-Noise Ratio (CNR), $(I_S - I_B) / \sigma_B$	52.34	3.53	63.08	3.35
Probe Concentration (μM), C	17	N.A.	17	N.A.
Noise-equivalent Concentration (NEC, μM), C / CNR	0.33	N.A.	0.27	N.A.

a.u. – arbitrary units. N.A. – not applicable.
