# Supplementary material for: Near-infrared photochromic miniprotein enables photoacoustic molecular imaging of acute inflammation and intracellular antigens
Source: Nat Methods. 2026 Oct 13;23(11):2274–85. doi: 10.1038/s41592-026-03255-y (PMC13736489; doi:10.1038/s41592-026-03255-y)
Supplement: Supplementary file 2 [file 41592_2026_3255_MOESM2_ESM.pdf]

nature portfolio

Corresponding author(s): Junjie Yao, Vladislav Verkhusha

Last updated by author(s): Aug 2, 2026

Reporting Summary

Nature Portfolio wishes to improve the reproducibility of the work that we publish. This form provides structure for consistency and transparency in reporting. For further information on Nature Portfolio policies, see our Editorial Policies and the Editorial Policy Checklist.

Statistics

For all statistical analyses, confirm that the following items are present in the figure legend, table legend, main text, or Methods section.

n/a	Confirmed	
☐	☒	The exact sample size (*n*) for each experimental group/condition, given as a discrete number and unit of measurement
☐	☒	A statement on whether measurements were taken from distinct samples or whether the same sample was measured repeatedly
☐	☒	The statistical test(s) used AND whether they are one- or two-sided<br>*Only common tests should be described solely by name; describe more complex techniques in the Methods section.*
☒	☐	A description of all covariates tested
☐	☒	A description of any assumptions or corrections, such as tests of normality and adjustment for multiple comparisons
☐	☒	A full description of the statistical parameters including central tendency (e.g. means) or other basic estimates (e.g. regression coefficient) AND variation (e.g. standard deviation) or associated estimates of uncertainty (e.g. confidence intervals)
☐	☒	For null hypothesis testing, the test statistic (e.g. *F*, *t*, *r*) with confidence intervals, effect sizes, degrees of freedom and *P* value noted<br>*Give P values as exact values whenever suitable.*
☒	☐	For Bayesian analysis, information on the choice of priors and Markov chain Monte Carlo settings
☒	☐	For hierarchical and complex designs, identification of the appropriate level for tests and full reporting of outcomes
☒	☐	Estimates of effect sizes (e.g. Cohen's *d*, Pearson's *r*), indicating how they were calculated

Our web collection on statistics for biologists contains articles on many of the points above.

Software and code

Policy information about availability of computer code

Data collection	Flow cytometry data were collected using BD Accuri C6 v.1.0.264.21. Fluorescence in cells was measured on a microscope controlled by SlideBook v.6.0.8 , images were acquired by Flash4.0 V3 camera (Hamamatsu), supported by DCAM-API.<br>PA and US data were collected by a hybrid 3D photoacoustic and ultrasound localization microscopy system. The hybrid system comprises a programmable ultrasound scanner (Vantage 256, Verasonics, Kirkland, WA), a multi-spectral pulsed laser for PA illumination, and a customized 2D semispherical ultrasound transducer array (Imasonics, France) for both PA and US imaging.<br>Fluorescence in animals was collected by a lab-made wide-field fluorescence imaging system used a high-sensitivity InGaAs camera that has a broad detection spectrum from 600nm to 1.7 µm (Ninox 640 II, Raptor Photonics). CODEX data acquisition was performed with Akoya PhenoCylcer Fusion 2.2.0 (Quanterix).
Data analysis	Data processing and statistical analyses were conducted using OriginPro 2021b (OriginLab) and Microsoft Excel v.15.36.<br>The Olympus IX81 microscope was operated with a SlideBook v.6.0.8 software (Intelligent Imaging Innovations).<br>The Flow cytometry data were analyzed using a FlowJo v.7.6.2 software.<br>PA and US data were processed by MATLAB R2024b.<br>All the statistical analysis for PA and US were conducted by GraphPad Prism.<br>CODEX data processing was performed with Akoya PhenoCylcer Fusion 2.2.0 and QuPath software. For CODEX single-cell segmentation SPACEc package was used. For CODEX cell-type analysis data were processed with scanpy and sklearn Python packages.

For manuscripts utilizing custom algorithms or software that are central to the research but not yet described in published literature, software must be made available to editors and reviewers. We strongly encourage code deposition in a community repository (e.g. GitHub). See the Nature Portfolio guidelines for submitting code & software for further information.

Data

Policy information about availability of data

All manuscripts must include a data availability statement. This statement should provide the following information, where applicable:

- Accession codes, unique identifiers, or web links for publicly available datasets
- A description of any restrictions on data availability
- For clinical datasets or third party data, please ensure that the statement adheres to our policy

The nucleotide sequences of rsmiRFPnano in GenBank hasaccession number PX270336. The image processing codes used in this study are available at Duke Photoacoustic Imaging Lab's GitLab page: https://gitlab.oit.duke.edu/pilab/blvra. The major plasmids constructed during this research, their maps and sequences are deposited to Addgene depository.

Research involving human participants, their data, or biological material

Policy information about studies with human participants or human data. See also policy information about sex, gender (identity/presentation), and sexual orientation and race, ethnicity and racism.

Reporting on sex and gender	Not relevant in this study
Reporting on race, ethnicity, or other socially relevant groupings	Not relevant in this study
Population characteristics	Not relevant in this study
Recruitment	Not relevant in this study
Ethics oversight	Not relevant in this study

Note that full information on the approval of the study protocol must also be provided in the manuscript.

Field-specific reporting

Please select the one below that is the best fit for your research. If you are not sure, read the appropriate sections before making your selection.

☒ Life sciences ☐ Behavioural & social sciences ☐ Ecological, evolutionary & environmental sciences

For a reference copy of the document with all sections, see nature.com/documents/nr-reporting-summary-flat.pdf

Life sciences study design

All studies must disclose on these points even when the disclosure is negative.

Sample size	Standard N>3 independent experiments were performed for most cases, unless noted in the figure legend. For flow cytometry at least 30,000 cells per sample were recorded. For animal experiments sample size included 3 mice for each experiment. Sample size was based on experience in prior studies.
Data exclusions	No data were excluded from analysis.
Replication	Usually three independent replications of the experiments were performed for all presented protein variants and labeled structures in different cells, different days of transfection and different days of recording. All attempts at replication were successful.
Randomization	Group allocation used in our study were randomly assigned to animals and cultured cells
Blinding	Blinding is not relevant to our studies because they involved test group versus control group for evaluation performance of the constructs and the data are not relevant to clinical trials.

Reporting for specific materials, systems and methods

We require information from authors about some types of materials, experimental systems and methods used in many studies. Here, indicate whether each material, system or method listed is relevant to your study. If you are not sure if a list item applies to your research, read the appropriate section before selecting a response.

Materials & experimental systems

n/a	Involved in the study
☐	☒ Antibodies
☐	☒ Eukaryotic cell lines
☒	☐ Palaeontology and archaeology
☐	☒ Animals and other organisms
☒	☐ Clinical data
☒	☐ Dual use research of concern
☒	☐ Plants

Methods

n/a	Involved in the study
☒	☐ ChIP-seq
☐	☒ Flow cytometry
☒	☐ MRI-based neuroimaging

Antibodies

Antibodies used

The CODEX antibodies panel is detailed in the supplemental table (easier to read) but included here as well:
RRID Company Antibody Clone
AB_2736987 BioXcell 16/32 2.4G2
AB_2572996 Invitrogen aSMA 1A4
AB_1107651 BioXcell B220 Ra3-6B2
AB_3097587 BioLegend BRG1 W22111B
AB_535944 BioLegend CD103 2E7
AB_393741 BD CD106 429 (MVCAM.A)
AB_393577 BD CD11b M1/70
AB_313770 BioLegend CD11c N418
AB_10959655 BioLegend CD138 281-2
NA BioRad CD169 MOMA-1
AB_395047 BD CD19 1D3
AB_2687821 BioXcell CD200 OX-90
AB_1236456 BioLegend CD27 LG.3A10
AB_395697 BD CD3 17A2
AB_394815 BD CD31 MEC 13.3
AB_2754555 BioXcell CD38 NIMR5
AB_393575 BD CD4 RM4-5
AB_394645 BD CD44 IM7
AB_1134214 BioLegend Ly6C HK1.4
AB_394206 BD Ly6G 1A8
AB_313316 BioLegend MHCII M5/114.15.2
NA BioXcell MPO 6G4
AB_1727467 BD NKp46 29A1.4
AB_1099464 BioXcell Tim3 RMT3-23
AB_10950158 BioXcell TCRb H57-597 (HB12)
AB_394606 BD CD45 30-F11
NA SinoBiological CD62L 414
AB_394742 BD CD71 C2F2
AB_1089066 BioLegend CD73 TY-11.8
AB_313144 BioLegend CD86 GL-1
AB_2275792 BD CD8a 53-6.7
AB_313168 BioLegend CD90 G7
AB_2869866 BD F4/80 T45-2342
AB_2102369 R&D FAP Polyclonal
AB_467576 eBioscience FOXP3 FJK-16s
AB_10828444 Miltenyi Biotech FR4 TH6
NA Novus GRZB AF1865
AB_392775 BD H-2Db 28-14-8
AB_313734 BioLegend H-2Kb AF6-88.5
AB_2566332 BD HDAC2 13G8C67
AB_568569 Invitrogen HIF-1a ESEE122
AB_394858 BD IgD 11-26c.2a
AB_531826 Hybridoma keratin8 TROMA-1
AB_10854564 Invitrogen Ki67 SolA15
AB_10949054 BioXcell KLRG1 2F1
AB_2291192 BioLegend PD-L1 10F.9G2
AB_1877086 BioLegend PD1 29F.1A12
AB_1089187 BioXcell Podoplanin 8.1.1
AB_313348 BioLegend Sca-1 D7

Validation

The tissue arrays were stained with the validated panels of CODEX antibodies that were validated using protocols in: https://doi.org/10.1038/s41596-021-00556-8 and demonstrated their use in tissues with expected staining patterns: https://doi.org/10.1016/j.celrep.2023.113494.

Eukaryotic cell lines

Policy information about cell lines and Sex and Gender in Research

Cell line source(s)	HeLa (CCL-2) and HEK293T (CRL-3216) cell lines were obtained from ATCC.
Authentication	Cell lines were not additionally authenticated.
Mycoplasma contamination	Cell lines were not additionally tested for mycoplasma.
Commonly misidentified lines (See ICLAC register)	No commonly misidentified cell lines were used.

Animals and other research organisms

Policy information about studies involving animals; ARRIVE guidelines recommended for reporting animal research, and Sex and Gender in Research

Laboratory animals	Animal experiments were performed using Blvra^{-}/$^{-}$ mice (8-12 weeks old, 20-30 g) . The founder animals of the Blvra^{-}/$^{-}$ strain were a gift from Drs. K.Terai and M.Matsuda (Kyoto University, Japan) and were used to establish an in-house breeding colony. All animals were maintained on a 12 h light/dark cycle in facility supplied with fresh air with humidity equivalent to outside ambient air.
Wild animals	No wild animals were used in the study.
Reporting on sex	All animals used were female mice (6-8 weeks old, 20-30g).
Field-collected samples	No field-collected samples were used in the study.
Ethics oversight	All live animal procedures were performed following the guidelines of the National Institutes of Health and were approved by the Institutional Animal Care and Use Committee at the Duke University.

Note that full information on the approval of the study protocol must also be provided in the manuscript.

Plants

Seed stocks	No seed stocks were used in this study
Novel plant genotypes	No plants were included in this study
Authentication	No relevant to study

Flow Cytometry

Plots

Confirm that:

☒ The axis labels state the marker and fluorochrome used (e.g. CD4-FITC).

☒ The axis scales are clearly visible. Include numbers along axes only for bottom left plot of group (a 'group' is an analysis of identical markers).

☒ All plots are contour plots with outliers or pseudocolor plots.

☒ A numerical value for number of cells or percentage (with statistics) is provided.

Methodology

Sample preparation	Prior to acquisition, cell pellets were washed with PBS and diluted in cold PBS to density 500,000 cells per ml .
Instrument	BD Accuri C6 flow cytometer.
Software	BD Accuri C6 v.1.0.264.21, FlowJo v.7.6.2.
Cell population abundance	At least 30,000 cells per sample were recorded.

Gating strategy	Initial gates - FSC-A/SSC-A to discriminate cells from debris; then cells were gated in FSC-W/FSC-A to discriminate single cells; then cells were gated in SSC-W/SSC-A to discriminate live cells. Mock transected cells were used for selecting negative population.

☒ Tick this box to confirm that a figure exemplifying the gating strategy is provided in the Supplementary Information.
